# The Effect of Fluoride Ions on Plasma Electrolytic Oxidation Coatings Formed on Magnesium Alloys

**DOI:** 10.3390/ma19153169

**Published:** 2026-07-24

**Authors:** Łukasz Florczak, Andrzej Sobkowiak

**Affiliations:** Department of Physical Chemistry, Faculty of Chemistry, Rzeszów University of Technology, 35-029 Rzeszów, Poland

**Keywords:** magnesium, conversion coatings, PEO, complex fluorides

## Abstract

**Highlights:**

- Complex fluoride ions significantly influence the formation of PEO coating on Mg alloys.
- Most complex fluorides hydrolyze in the PEO process environment depending on the pH of the electrolyte and the presence of stabilizing additives.
- Preventing hydrolysis of complex fluoride ions is the crucial process enabling higher fluorine incorporation and improving coating morphology, phase composition, and corrosion resistance.

**Abstract:**

Plasma electrolytic oxidation (PEO) is an effective method for developing protective conversion coatings on magnesium and its alloys. The efficiency of this process is governed by several factors, including electrical parameters and electrolyte composition. Typically, PEO has been performed in alkaline silicate or phosphate solutions, often enriched with simple fluoride ions (F^−^) to improve the mechanical and anticorrosive properties of the resulting layers. Recently, the positive impact of complex fluoride ions (such as ZrF_6_^2−^, TiF_6_^2−^, SiF_6_^2−^, AlF_6_^3−^ and PF_6_^−^) on the properties of conversion coatings on magnesium substrates has been shown. This review analyzes how these complex precursors influence the phase composition, morphology, and corrosion resistance of coatings. Furthermore, these parameters are compared with those obtained by using electrolytes composed of a mixture of simple fluorides and additional constituents that provide the incorporation of the appropriate elements into the coating structure (phosphorus, silicon, zirconium, titanium, or aluminum). It was indicated that when using complex fluoride salts, an important aspect is the stability of the electrolyte, which depends on pH and the presence of stabilizing additives. Ensuring that the decomposition of the complex fluoride occurs during the PEO process increases the amount of fluorine incorporated into the layers formed, leading to the formation of a coating with enhanced properties.

## 1. Introduction

Magnesium alloys are among the lightest structural materials, which makes them widely used in the automotive and aerospace industries and due to their biocompatibility and biodegradability, also in the biomedical field [1,2,3,4]. However, their primary disadvantage is caused by their high susceptibility to corrosion in humid conditions. Although research on methods for improving the properties of magnesium alloys has been ongoing for a long time, there is still a constant need for further development of this field [5,6,7,8,9]. In addition to developing new alloy compositions, designing customized heat treatments, and the use of corrosion inhibitors, methods to create surface protection for magnesium and its alloys are being explored extensively [10,11,12,13,14].

Plasma electrolytic oxidation (PEO) represents an effective method of producing protective conversion coatings on light metals, including magnesium, aluminum, and titanium [15,16,17]. The properties of the resulting layer are influenced by a variety of factors. The primary category includes electrical parameters, such as the power supply mode (direct current, alternating current, or pulsed mono/bipolar), current density, final voltage, and pulse waveform (frequency, duty cycle, and anode-to-cathode signal ratio). In addition, the electrolyte composition determines the chemistry of the resulting conversion coating. Since PEO is a dynamic process characterized by changes in the discharge regime (non-spark, spark, micro-arc, and large arc), the final morphology of the coating also depends on the substrate material, its pretreatment, and the total processing time [18,19,20,21].

Magnesium alloys are the second most common substrate material for PEO, following aluminum alloys, and research on this system remains intensive [22,23,24]. Contemporary research on PEO of magnesium and its alloys often involves pre- and post-treatment processes of the substrate to improve biocompatibility or seal the porous coating [25,26,27,28,29]. Furthermore, PEO coatings have been increasingly used as a primer for organic top layers, forming duplex coating systems [30,31,32,33]. However, there is still interest in the modification of electrolytes used for PEO by introducing new additives, including inert nanoparticles and corrosion inhibitors [13,34,35,36].

Among various modifications, the introduction of fluoride ions is highly effective due to the formation of magnesium fluoride (MgF_2_, *K*_sp_ = 5.16 × 10^−11^). Although its solubility product is similar to that of magnesium hydroxide (Mg(OH)_2_, *K*_sp_ = 5.61 × 10^−12^) [37], MgF_2_ exhibits far superior chemical stability and lower dissolution rates in neutral and weakly acidic environments. Furthermore, from a thermodynamic perspective, MgF_2_ formation is highly favored, as evidenced by its extremely negative standard Gibbs free energy of formation (ΔG^0^ = −1071.1 kJ/mol), which provides exceptional structural stability compared to MgO (ΔG^0^ = −569.3 kJ/mol) and Mg(OH)_2_ (ΔG^0^ = −833.5 kJ/mol) [37]. This thermodynamic and kinetic stability promotes the rapid growth of a dense MgF_2_ barrier layer at the substrate interface and substantially improves corrosion resistance. However, free fluoride ions (F^−^) cause serious environmental and safety hazards due to their high toxicity. The use of complex fluoride salts can eliminate the presence of F^−^ ions in the electrolyte and release them in the plasma environment during the PEO process, ensuring the successful incorporation of fluorine into the coating structure.

The addition of complex fluorides represents a particularly noteworthy case [38,39]. The present work evaluates the rationale for the use of complex fluoride salts, especially ZrF_6_^2−^, TiF_6_^2−^, SiF_6_^2−^, and AlF_6_^3−^, which can undergo hydrolysis in aqueous environments and/or plasma-induced decomposition, leading to the liberation of simple F^−^ ions and also incorporating the remaining components of the additive into the conversion coating. Furthermore, this study identifies critical factors in the PEO process that involve complex fluorides and proposes future research directions for the application of fluoride additives in electrolytes designed for magnesium and its alloys.

## 2. Plasma Electrolytic Oxidation of Magnesium Substrates

**Figure 1:**
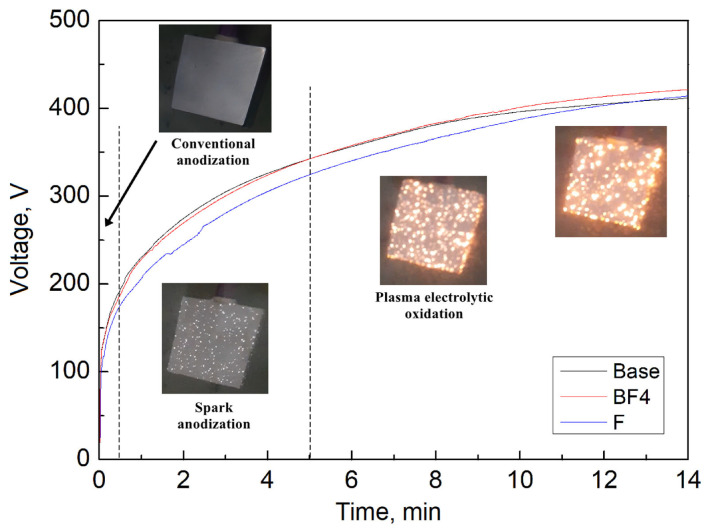
Voltage–time dependence during the PEO process in galvanostatic mode for different electrolytes [40].

Plasma Electrolytic Oxidation (PEO), also known as Micro Arc Oxidation (MAO), is a high-voltage anodizing technique used to produce conversion layers on magnesium and its alloys, among other substrates. The process is conducted under constant current (galvanostatic) or constant voltage (voltastatic) conditions. In the galvanostatic mode, as a result of the formation of an oxide insulation layer on the treated surface, an increase in applied voltage is required to maintain a preset constant current density. The PEO treatment always begins with a conventional non-sparking stage, followed by the onset of the first spark discharges (fine and white) upon reaching the breakdown voltage, at which point the process enters the spark oxidation stage and the rate of voltage increase diminishes. Subsequently, the discharge characteristics evolve: the sparks become more intense and less frequent, shifting to a yellow-orange color, and producing a characteristic acoustic signature. This takes place once the critical voltage is reached. It is widely recognized that the most significant structural changes in the conversion layer occur during this stage due to the extensive incorporation of electrolyte-derived species. Figure 1 illustrates the voltage–time curve and the surface appearance of the magnesium substrate during galvanostatic PEO treatment.

During electric discharges at high temperature (on the order of several thousand degrees), the substrate material melts and interacts with the electrolyte components. After being cooled, a coating composed of a mixture of substrate elements and electrolyte components is formed. Hussein et al. [41,42,43] have described three types of discharges: discharges at the coating–electrolyte interface at the top of the layer (type A), intense microdischarges that occur deep near the metal–coating interface (type B), and discharges within the micropores at the coating–electrolyte interface (type C). Cheng et al. [44] have expanded this model, distinguishing two other types of discharges: occurring in large pores near the interface between the inner and outer layers (type D) and forming pancake-shaped structures mainly within the outer coating layer (type E).

The PEO process is typically performed in an alkaline environment, which facilitates preliminary surface passivation, thereby enabling the onset of the plasma stage. The final products consist mainly of MgO and Mg(OH)_2_. To enhance the properties of the resulting coatings, other salts are added to the solution, most often silicates, phosphates, aluminates, and simple fluorides, allowing the synthesis of the corresponding magnesium compounds during PEO (Equations (1)–(8)) [22]:

Mg → Mg^2+^ + 2e^−^ (1)

Mg^2+^ + 2OH^−^ → Mg(OH)_2_ (2)

Mg(OH)_2_ → MgO + H_2_O (3)

2Mg^2+^ + SiO_3_^2−^ + 2OH^−^ → Mg_2_SiO_4_ + H_2_O (4)

Mg^2+^ + SiO_3_^2−^ → MgSiO_3_ (5)

3Mg^2+^ + 2PO_4_^3−^ → Mg_3_(PO_4_)_2_ (6)

Mg^2+^ + 2AlO_2_^−^ → MgAl_2_O_4_ (7)

Mg^2+^ + 2F^−^ → MgF_2_ (8)

Furthermore, in an aqueous alkaline environment, a significant amount of oxygen is generated throughout the plasma process, which can directly lead to the formation of MgO through a high-temperature reaction with the substrate (Equations (9) and (10)) [45]:

2H_2_O → 2H_2_ + O_2_ (9)

2Mg + O_2_ → 2MgO (10)

Different electrolyte additives are often used simultaneously to achieve a synergistic effect. Moreover, one of the approaches to primarily enhance the mechanical properties of a protective layer involves the addition of nanoparticles. During the PEO process, these nanoparticles can be melted and mixed with MgO or incorporated as intact particles into the coating structure. It has been shown [44] that type B discharges lead to the melting of nanoparticles, while types A and C result in the incorporation of entire particles. Thus, the form of the additive (dissolved salts or nanoparticle suspensions) can influence the mechanism of incorporation into the growing conversion coating structure.

## 3. Patents and Commercial Applications of PEO on Magnesium Substrates Using Fluoride-Containing Electrolytes

The application of fluorides in baths for the electrochemical synthesis of conversion layers on magnesium and its alloys has been documented in a wide range of patents and remains essential in several commercial technologies (Table 1).

As early as 1926, Keeler patented the use of hydrofluoric acid to produce a protective coating on magnesium through a low-voltage anodizing process [46]. Subsequent patents expanded the use of fluorine sources to include both simple and complex fluoride salts. Early commercial methods utilized simple fluoride compounds in low-voltage treatments [67]. Developed by Dow Chemical Company, the Dow 17 process involved an acidic electrolyte (pH = 5) consisting of chromates, phosphates, and fluorides, specifically ammonium bifluoride (NH_4_HF_2_) [51]. Meanwhile, the HAE process (created by Harry A. Evangelides) used a highly basic electrolyte (pH = 14) containing a mixture of potassium and aluminum hydroxides, as well as phosphate, manganate and fluoride in the form of KF [50]. Other companies holding patents for the production of conversion coatings on magnesium using simple fluorides were Ube Industries Ltd. (Japan) [53] and Electro Chemical Engineering GmbH (Switzerland) [54]. It is worth noting that the possibility of using complex fluoride compounds (H_2_SiF_6_, HBF_4_) was claimed as early as 1943 by Dow Chemical Company [48] and Buzzard [49] in their patent concerning Na_2_SiF_6_. Furthermore, Henkel, in its 2003 patent on low-voltage oxidation of light metals, included claims for a very broad list of complex fluorides which contained H_2_TiF_6_, H_2_ZrF_6_, H_2_HfF_6_, H_2_SiF_6_, H_2_GeF_6_, H_2_SnF_6_, H_3_AlF_6_, and HBF_4_, as well as their salts (both fully and partially neutralized) and mixtures thereof [58].

In commercial high-voltage (PEO) technologies, the use of fluorides in electrolytic baths was also common. The Tagnite process (Technology Applications Group) employed an alkaline silicate solution with fluoride or fluorosilicate additives [55]. Similarly, Magoxid-Coat [56] and Anomag [57] technologies reported to use fluorides [5]. Recent patents in the field of PEO disclosed the use of alkaline baths containing a mixture of complex and simple fluorides, together with organic compounds such as citrates [60], urea [61], and glycerol [66], which suggested their significant role in stabilizing the electrolyte.

Obviously, not all technologies contain fluorine compounds; e.g., Keronite did not claim this addition in its 2005 patent [68]. It should also be noted that many modern technologies (and their variants) remain undisclosed and operate as proprietary know-how rather than patented processes. Examples of contemporary technologies are Curtiss-Wright Surface Technologies (Keronite), Aalberts Surface Technologies GmbH (Magoxid-Coat), Technology Applications Group (Tagnite), IBC Coatings (CeraTough), Whyco Finishing Technologies (CeraFuse), and Micron Coating Group (OX-UHA Magnesium PEO).

The described changes in electrolytes used in the patented PEO processes are aimed at improving the properties of the protective layers and adapting their properties to technological requirements. However, the information on the subject is rather limited. Nevertheless, the persistent presence of complex fluoride salts in recently published patents indicates the desirable effect of these salts on the properties of the obtained coatings. A detailed description of the effects of the salts on the nature of the layers is discussed later.

## 4. Electrolytic Baths Containing Fluorides for PEO of Magnesium Substrates

### 4.1. Simple Fluorides

Simple fluorides, such as NaF and KF, are commonly used as additives in alkaline electrolytes for the PEO of magnesium alloys. It has been demonstrated [69] that the introduction of 8 g/L KF into an alkaline silicate electrolyte (1 g/L KOH + 10 g/L Na_2_SiO_3_) improved the properties of the resulting coating. The PEO process was performed on AM60B alloy using an AC signal (*j* = 6 A/dm^2^, *i*_a_/*i*_c_ = 1, *t* = 30 min). The presence of KF has increased the electrolyte conductivity (from 12.9 to 19.1 mS/cm), leading to a decrease in the work and final voltages (both positive and negative) necessary to maintain the applied current density. Consequently, this resulted in a reduction in the outer pore size (from 14 to 5 µm) and the thickness of the coating (from 37 to 32 µm). XRD analysis confirmed the presence of MgO, Mg_2_SiO_4_, and MgAl_2_O_4_ in the coating, with the additional presence of MgF_2_ and KMgF_3_ after the introduction of KF. These changes in the coating composition caused an increase in hardness (from ~500 to ~600 HV) and wear resistance (the wear rate decreased in a range from 1.05 to 0.684 × 10^−5^ mm^3^·N^−1^·m^−1^). The values recorded for the uncoated alloy were ~90 HV and 3.81 × 10^−4^ mm^3^·N^−1^·m^−1^, respectively.

The effect of KF addition in the range of 2 to 8 g/L on an alkaline silicate electrolyte (4 g/L KOH + 20 g/L Na_2_SiO_3_ + 10 mL/L ethylene glycol) on the PEO of AZ31 alloy under DC at constant voltage (*U* = 300 V, *t* = 30 min) has also been investigated [70]. EDS analysis showed that the main components of the coating were O, Mg, and Si (approximately 48, 30, and 15 at.%, respectively). Incorporation of C, Al, and F was also recorded. The fluorine content increased proportionally with its concentration in the bath, ranging from 1 to 3 at.%. XRD analysis has indicated the presence of MgSiO_3_, Mg_2_SiO_4_, and MgC_2_ in all coatings. In the case of electrolytes containing fluorine, the presence of MgF_2_ was also found. An increase in the diameters of the outer pores was observed as the fluorine concentration increased, which is opposite to the results reported in [69]. Nevertheless, corrosion tests (PDP, EIS in 3.5 wt.% NaCl) demonstrated that the coating produced with the addition of 8 g/L KF exhibited the best anti-corrosion properties. This behavior was attributed to the formation of the highest amount of MgF_2_.

The increase in KF concentrations has caused a significant increase in the thickness of the coating, from 5.84 µm without KF to 9.49, 25.61, and 27.83 µm for 5, 10, and 15 g/L of KF, respectively [71], which was accompanied by an increase in the size of the outer pores. The PEO process was performed using a magnesium alloy AZ31 in an alkaline phosphate electrolyte (2 g/L NaOH + 3 g/L (NaPO_3_)_6_) using a bipolar pulse signal in constant current mode (*j* = unknown, *f*_+_ = 1000 Hz, *D*_+_ = 40%, *f*_−_ = 120 Hz, *D*_−_ = 20%, *t* = 10 min). EDS measurements indicated the incorporation of both phosphorus and fluorine into the coatings formed. The fluorine content was equal to 7.67, 15.48, and 25.74 wt.% for the coatings produced in electrolytes with 5, 10, and 15 g/L KF, respectively. XRD analysis showed the presence of crystalline MgF_2_ together with MgO in the coatings formed at KF concentrations of 10 and 15 g/L. Based on the corrosion tests performed (PDP, EIS, hydrogen evolution, and immersion test in SBF), the coating produced with the highest KF concentration (15 g/L) was found to be the most corrosion-resistant. Additionally, in vitro cytotoxicity tests have indicated that the fluorine-containing coating exhibited cytocompatibility and did not have a cytotoxic effect on L-929 cells.

The influence of fluorine ions present in the PEO coating on cytocompatibility has also been examined [72], where the coatings were prepared on AZ31 alloy in an alkaline silicate electrolyte (0.1 mol/L KOH + 0.4 mol/L Na_2_SiO_3_·9H_2_O) with the addition of 0.05–0.2 mol/L KF·2H_2_O (corresponding to 2.9–11.6 g/L KF). The PEO process was carried out under constant current density conditions (*j* = 5 A/dm^2^, *f* = 300 Hz, *D* = 10%, *t* = 15 min). The fluorine content reached 1.85, 4.72, and 13.5 at.% for KF concentrations in the electrolyte equal to 0.05, 0.1, and 0.2 mol/L, respectively. The main component of the coating was MgO. In this research, a series of studies on corrosion behavior were carried out in vitro in SBF (PDP, local alkalization, hydrogen evolution, and immersion test) and in vivo (subcutaneous implantation in mice). Additionally, in vitro biocompatibility was evaluated through the cultivation of osteoblast-like cells MC3T3-E1, together with evaluations of cytotoxicity, cell adhesion, cell proliferation, live/dead cell staining, and the hemolysis ratio. It was shown that fluorine incorporation did not induce significant cytotoxicity and cell adhesion was found to be enhanced on the coating surface. The coating with a low fluorine content promoted cell growth on its surface, whereas the coating with a high fluorine content resulted in a slight inhibition of cell growth. Overall, the good cytocompatibility of the fluorine-containing coating was found, characterized by the absence of cytotoxic effects and the improvement in cell adhesion and proliferation.

The addition of NaF in the range of 0.1–0.5 mol/L (i.e., 4.2–21.0 g/L) to an alkaline silicate-phosphate electrolyte (0.7 mol/L NaOH + 0.15 mol/L Na_3_PO_4_ + 0.175 mol/L Na_2_SiO_3_) has caused an improvement in the properties of the conversion layer formed on AZ31 magnesium alloy [73]. The PEO process was conducted using a monopolar pulsed current (*j* = unknown, *f* = 2200 Hz, *D* = 44%, *t* = 10 min). The enhancement of the mechanical properties of the synthesized coatings was observed with the increase in the fluorine content in the electrolyte. A slight increase in the coating thickness (from ~28 µm without the additive to ~32 µm with 0.5 mol/L NaF) and roughness (an increase in the *R*_a_ coefficient from ~2 to ~3 µm) was recorded in the work. The hardness was increased for both the outer layer (from ~530 to ~590 HV) and the inner layer (from ~430 to ~500 HV) after the introduction of 0.5 mol/L NaF.

As follows from the PEO carried out on AZ91D alloy with the addition of KF in the range of 0.01–1.00 mol/L (0.58–58.10 g/L), the increase in the concentration of fluoride ions in the electrolyte does not always have a positive effect on the layer formed [74]. In the PEO process, an alternating voltage signal was used (*U* = ±100 V, *f* = 50 Hz, *t* = 15/60 min) and the base electrolyte consisted of an alkaline silicate solution (0.04 mol/L KOH + 0.05 mol/L Na_2_SiO_3_·5H_2_O (electrolyte A) or 0.05 mol/L KOH + 0.15 mol/L Na_2_SiO_3_·5H_2_O (electrolyte B)). Even a small addition of KF (0.02 mol/L = 1.16 g/L) to electrolyte A caused a significant increase in coating thickness (from ~22 µm to ~60 µm) after 60 min of oxidation. For the 15 min oxidation process, coating growth was observed up to a KF content of 0.2 mol/L (from 20 to 45 µm). However, the increase in the KF concentration to 1 mol/L (58.1 g/L) decreased the adhesion of the coating to the magnesium substrate and the coating became more porous. An uneven incorporation of fluorine into the coating was found, with a significant increase in its concentration near the magnesium substrate. XRD measurements indicated that the coating was mainly composed of Mg_2_SiO_4_. The presence of crystalline MgF_2_ was not detected. A mechanism of incorporation of fluoride ions into the conversion coating was proposed, according to which fluoride anions are initially chemisorbed on the metal surface (Mg|F_ads_^−^) and subsequently form a barrier layer composed of MgF_2_. After micro-discharges appeared, this layer was converted to MgO through the exchange of fluorine with oxygen atoms derived from the electrolyte. The fluorine released remained in the vicinity of the metal and participated in the formation of an amorphous phase. Corrosion tests (PDP in 2% NaCl) indicated that the best protective properties were demonstrated by coatings produced in the presence of 0.02–0.05 mol/L KF in the bath (*j*_corr_ = 31.6–12.3 µA/cm^2^, in electrolyte A, and *j*_corr_ = 1.04–2.37 µA/cm^2^ in electrolyte B). SEM images showed that coatings formed at the higher silicate concentration (0.15 mol/L) exhibited a larger number of internal pores; however, they had better corrosion resistance, confirming that the inner barrier layer of PEO coatings is crucial in this aspect.

Structural changes in the inner layer of PEO coatings resulting from the introduction of NaF (0.4–2.0 g/L) into the electrolyte have been described [75] in a series of PEO syntheses on AZ31 alloy using a pulsed current signal (*j* = 4.3 A/dm^2^, *f* = 100 Hz, *D* = 10%, *t* = 6 min) in an alkaline silicate-phosphate electrolyte (1 g/L NaOH + 12 g/L Na_2_SiO_3_ + 6 g/L Na_3_PO_4_). SEM and EDS analyses did not detect the presence of fluorine near the substrate. However, HAADF-STEM measurements indicated that after the addition of NaF to the electrolyte, a crystalline MgF_2_ phase (containing both rutile and cubic phases) formed near the magnesium substrate together with MgO. The addition of 0.8–2.0 g/L NaF led to the formation of a continuous, nano-sized, thin fluorine-containing nanolayer (FNL) with a thickness of 100–200 nm. Based on electrochemical corrosion tests (PDP, EIS in 0.1 mol/L NaCl), a significant improvement in barrier properties was found for the coatings synthesized with a NaF content of 0.8–2.0 g/L. The impedance measurements showed an increase in the impedance values for the time constant at the lowest frequencies, which was interpreted as a contribution from the formed FNL. The best anti-corrosion properties were exhibited by the coating with the addition of 2 g/L NaF, despite the fact that the coating with 0.8 g/L NaF was characterized by the lowest outer porosity, which confirms the crucial role of the inner layer in maintaining corrosion resistance.

A comparison of the properties of conversion coatings produced with the addition of NaF or KF (1–6 g/L) has been carried out [76] using a magnesium alloy AZ41, under identical electrical conditions (bipolar pulse current mode *j* = 13 A/dm^2^, *i*_a_/*i*_c_ = 2, *f* = 50 Hz, *t* = 60 min), in an alkaline silicate electrolyte (4 g/L KOH + 6 mL/L water glass (24.1–35.0 wt.% SiO_2_ + 8.7–12.3 wt.% Na_2_O)). Corrosion tests (PDP in 3% NaCl) demonstrated that the best anti-corrosion properties were obtained by the coating produced with the addition of 5 g/L KF (*j*_corr_ = 0.04 µA/cm^2^). In the case of the NaF additive, the lowest corrosion current densities were obtained at a concentration of 6 g/L (*j*_corr_ = 0.07 µA/cm^2^). The advantage of KF over NaF was attributed to the higher electrical mobility of K^+^ ions compared to that of Na^+^ ions. However, the study did not specify either the thickness of the coatings or the fluorine content within the layers.

Although KF and NaF are the most commonly used simple fluoride additives to electrolytes in the PEO of magnesium alloys, attempts have also been made to use other fluorides. The effect of the addition of NH_4_F has been compared with that of KF [77]. The PEO process was carried out on AZ31 alloy in an alkaline silicate electrolyte (5 g/L NaOH + 15 g/L Na_2_SiO_3_·9H_2_O) containing the investigated fluorides at concentrations ranging from 0.042 to 0.216 mol/L. Synthesis was carried out using a pulsed unipolar current in constant current mode (*j* = 2 A/dm^2^, *f* = 500 Hz, *D* = 20%, *t* = 15 min). The addition of KF to the electrolyte caused a systematic increase in electrolyte conductivity (from 42.7 to 57.9 mS/cm) and pH (from 12.9 to 13.3). On the contrary, the introduction of NH_4_F decreased both conductivity (from 34.6 to 19.2 mS/cm) and pH (from 12.6 to 10.3). The presence of NH_4_F (0.042–0.216 mol/L) significantly increased the thickness of the coatings formed in the range of 25.8–44.8 µm; the effect in the case of KF was less pronounced, 25.0–28.9 µm. SEM analysis showed that the structure of the coatings prepared in the presence of KF was more homogeneous, whereas a large number of internal pores could be found in the coatings produced with the addition of NH_4_F. However, a significantly higher fluorine content was detected in the coatings produced in the presence of NH_4_F (ranging from 1.39 to 20.91 wt.%) compared to those obtained from the KF bath (0.94–5.43 wt.%). XRD analysis indicated that the coatings synthesized with the addition of KF were mainly composed of MgO and a smaller amount of Mg_2_SiO_4_, while the presence of crystalline MgF_2_ was not observed. For the coatings obtained from the electrolyte containing NH_4_F in addition to the peaks corresponding to MgO and Mg_2_SiO_4_, a peak corresponding to crystalline MgF_2_ was recorded only at concentrations of 0.158 and 0.216 mol/L. This observation was explained by the fact that, taking into account the solubility constants of Mg(OH)_2_ and MgF_2_, only for these two cases did the concentrations of OH^−^ and F^−^ ions in the electrolyte favor the formation of MgF_2_. Corrosion tests (PDP in 3.5 wt.% NaCl) have shown that the coating produced with 0.216 mol/L NH_4_F (8 g/L) exhibited the best anti-corrosion properties.

In the presence of 0.46 mol/L (1.19 g/L) LiF in an alkaline silicate electrolyte (0.15 mol/L KOH + 0.086 mol/L Na_2_SiO_3_), the obtained coating is less resistant to corrosion (EIS in 0.1 mol/L NaCl and PBS) than the coatings obtained with the addition of NaF or Na_2_SiF_6_ in an equimolar amount of fluorine [78]. It was suggested that the effect was caused by the small amount of fluorine incorporated due to the poor solubility of LiF (1.34 g/L at 20 °C). The PEO process was carried out on AZ31 alloy using a bipolar current in galvanostatic mode (*j* = 17.5 A/dm^2^, *U*_+_ = 400 V, *U*_−_ = −30 V, *f* = 67 Hz, *D* = 66.7%, *t* = 4/30 min). Details of this study are described in Section 4.2.3 during the discussion on the effect of Na_2_SiF_6_.

The effect of the addition of CaF_2_ (3 g/L) to an alkaline phosphate electrolyte (2 g/L KOH + 5 g/L K_3_PO_4_·3H_2_O) has been investigated [79] during the PEO process of AZ91 alloy with the use of a pulsed DC current (*j* = 10 A/dm^2^, *D* = 25%). Incorporation of both F (2.38 wt.%) and Ca (6.19 wt.%) was detected. The fluorine-containing coating was thicker and had fewer pores, although with larger diameters. XRD measurements indicated the presence of crystalline MgF_2_. The introduction of fluorine to the coating led to an increase in corrosion resistance (PDP, EIS in SBF). This is an interesting observation because, in contrast to NH_4_F, KF, and NaF, CaF_2_ is practically insoluble in water (0.016 g/L at 20 °C); nevertheless, fluorine was successfully incorporated into the coating.

Only a few studies have demonstrated PEO synthesis in acidic electrolytes containing simple fluorides. In [80], an acidic fluoride electrolyte (15 g/L KF + 2 g/L tartaric acid (C_4_H_6_O_6_)) was used with the addition of 3–5 g/L KH_2_PO_4_ at pH 5.8–6.2. The PEO process was performed on AZ91D alloy using monopolar and bipolar signals (*j* = 2 A/dm^2^, *f* = 500 Hz, *D*_+_ = 50%, *D*_−_ = 30%, *t* = 60 min). The coating produced using the monopolar signal without KH_2_PO_4_ was loose and porous. The introduction of KH_2_PO_4_ caused the coating to become denser. The switch of the signal from monopolar to bipolar led to the formation of an even more compact morphology. XRD analysis has shown that the coating produced without KH_2_PO_4_ was mainly composed of MgO, together with small amounts of MgF_2_ and KMgF_3_. The application of the monopolar signal in the presence of KH_2_PO_4_ resulted in the incorporation of a greater amount of MgF_2_ and KMgF_3_. In turn, the use of the bipolar signal led to the formation of the coating with the highest KMgF_3_ content. XPS measurements also indicated the presence of magnesium phosphates (Mg_3_(PO_4_)_2_ and MgHPO_4_) in the coatings prepared in the presence of KH_2_PO_4_ in the electrolyte, which was related to a decrease in the MgO content in the layer. Corrosion tests (PDP, EIS in 3.5 wt.% NaCl) have shown that the coating produced with the phosphate additive under the bipolar signal exhibited the best protective properties. This coating also maintained long-term corrosion resistance, as pitting corrosion occurred only after 216 h of immersion.

### 4.2. Complex Fluorides

#### 4.2.1. ZrF_6_^2−^

Potassium hexafluorozirconate is the complex fluoride salt most frequently used in the PEO of magnesium and its alloys. This compound has been employed in both acidic and alkaline electrolytes.

An acidic electrolyte containing 3 g/L K_2_ZrF_6_ and a mixture of H_3_PO_4_ and NaH_2_PO_4_ with pH 4–5 has been used for the PEO of AZ91D alloy [81]. Syntheses were carried out using a monopolar signal (*D* = 50%). The effects of electrical conditions (current density and frequency) and synthesis time on the anti-corrosion properties and thermal stability of the produced layers were investigated. It was shown that increasing the frequency (from 10 to 1000 Hz) and decreasing the current density (from 6.6 to 3.3 A/dm^2^) as well as the electrolysis time (from 30 to 10 min) improved the thermal shock resistance. The coating synthesized at a frequency of 1000 Hz exhibited the best anti-corrosion properties (PDP). XRD analysis indicated that the coating was mainly composed of *t*-ZrO_2_ (tetragonal) and a small amount of *c*-ZrO_2_ (cubic). Interestingly, the presence of MgO, which was typical for PEO coatings produced in alkaline electrolytes, was not detected.

Magnesium and its alloys are highly susceptible to dissolution in acidic media; therefore, methods to overcome this limitation have been investigated. A series of PEO syntheses was performed on AZ31 alloy using a direct current (*U* = 490 V, *t* = 30 min) in acidic solutions containing 0.025 mol/L (7.1 g/L) K_2_ZrF_6_ [82]. The effects of three phosphate additives, H_3_PO_4_, NaH_2_PO_4_, and Na_2_HPO_4_, on the electrolyte stability, discharge characteristics, and properties of the PEO coatings were analyzed. The mole ratio of K_2_ZrF_6_ to each additive was 9:1. Based on the observation of changes in pH and conductivity values of solutions before and after the PEO process, as well as the evaluation of the transparency of the electrolyte, it was found that the K_2_ZrF_6_ electrolyte with NaH_2_PO_4_ addition exhibited the best stability. The coating obtained in this electrolyte was characterized by a favorable microstructure and high corrosion resistance. It was also indicated that in the electrolyte containing NaH_2_PO_4_, the PEO process reached the plasma stage the fastest and the discharges themselves were the most stable and uniform, which was manifested by minor current fluctuations during oxidation. The coatings produced in an acidic environment consisted of three layers: a loose outer layer, a transition layer, and a compact inner layer. It is worth noting that the produced coatings contained large amounts of fluorine across all layers. The inner layer of the coating prepared with the addition of NaH_2_PO_4_ contained as much as 51.4 at.% F, as well as 21.9, 18.5, and 8.1 at.% of Mg, O, and Zr, respectively, and the fluorine content in the transition layer and the outer layer was equal to 49.9 and 47.4 at.%, respectively. XRD analysis has shown that the synthesized coatings were composed of *m*-ZrO_2_ (monoclinic), *t*-ZrO_2_ (tetragonal), and MgF_2_. It was suggested that due to the hydrolysis of Zr^4+^ ions, H_3_PO_4_ should be the most effective among the additives applied in stabilizing electrolytes. However, as a relatively strong acid (pH = 2.3), it caused an intensive dissolution of the magnesium alloy. Therefore, the addition of NaH_2_PO_4_ (pH = 3.7) provided the best electrolyte stabilizing effect, preventing both the hydrolysis of Zr^4+^ ions and the degradation of magnesium alloy.

The acidic base electrolyte consisting of 10 g/L K_2_ZrF_6_, 8 g/L NH_4_H_2_PO_4_, 3 g/L KF, and 5 g/L trisodium citrate (C_6_H_5_O_7_Na_3_) has also been used in the PEO of AM50 alloy under pulsed DC (*U* = 400 V, *f* = 500 Hz, *D* = 50%, *t* = 10 min) [83]. The coating obtained in this electrolyte exhibited the best anti-corrosion properties, which was attributed to the completely filled pores. EDS analysis indicated that the interior of the large pores contained basically Mg and F (41.3 and 35.6 wt.%, respectively), whereas the structure surrounding the pores was mainly composed of Zr, Mg, and O (37.1, 21.0, and 19.4 wt.%, respectively). XRD analysis indicated that the coating produced in the base electrolyte mainly contained *t*-ZrO_2_, Mg_2_Zr_5_O_12_, as well as small amounts of MgO and MgF_2_. Changes in the electrolyte composition resulted in visible differences in the structure of the obtained coatings and their anti-corrosion properties. The removal of NH_4_H_2_PO_4_ from the electrolyte had the greatest impact on the decrease in anti-corrosion properties of the coating, which confirms the crucial role of the additive in the stabilization process and the inhibition of local corrosion. The coating produced without this additive differed significantly in surface morphology, showing the absence of the characteristic porous structure. In turn, the removal of KF from the bath caused the disappearance of MgF_2_ responsible for the pore-sealing structure, which also reduced the anti-corrosion properties. In the absence of trisodium citrate in the solution, the size of the outer pores increased significantly and the inner layer became less compact. Decreasing the concentration of K_2_ZrF_6_ from 10 to 3 g/L led to the formation of larger pores in the outer layer and numerous internal pores at the interface of the outer porous layer and the inner dense layer.

In subsequent research [84] the effect of the addition of a simple fluoride salt (8 g/L KF) to an acidic electrolyte of 10 g/L K_2_ZrF_6_, 8 g/L NH_4_H_2_PO_4_, and 5 g/L trisodium citrate on the coatings obtained by the PEO process of AM50 alloy using a pulsed DC (*U* = 400 V, *f* = 500 Hz, *D* = 50%, *t* = 10 min) has been investigated. The coating growth process was described, taking into account phenomena that occur even at low voltage values before the beginning of spark discharge occurrence. In the absence of KF, all electrolyte components (O, Zr, F, and P) were found to be incorporated into the layer that formed at this stage. The presence of KF in the electrolyte resulted in the appearance of an additional area with a different morphology and composition, rich in fluorine and also magnesium and aluminum from the substrate, which indicated the local formation of MgF_2_ even before the appearance of discharges. It was established that the introduction of KF to the electrolyte decreased the anodic dissolution of magnesium during the pre-discharge stage and enabled the faster formation of a thin layer containing MgF_2_, stabilizing the surface before reaching the plasma stage. The addition of KF accelerated the coating formation and the oxide layer appeared at a voltage lower than that in the KF-free electrolyte. Regardless of the presence of KF in the electrolyte after discharges began, the surface morphology changed from a flat, cracked structure to a porous structure typical for PEO. However, during the plasma stage, the presence of KF led to faster coating growth and the final thickness increased from 17 to 25 µm. It was observed that the large pores were filled with MgF_2_ as the oxidation time increased. This was caused by the differences between the solidifying points of the coating components, equal to 1266, 2680, and 2825 °C for MgF_2_, ZrO_2_, and MgO, respectively; therefore, MgF_2_ was deposited last and sealed the pores. It was also suggested that K_2_ZrF_6_ was primarily a source of Zr, which was incorporated into the coating in the form of ZrO_2_ and Mg_2_Zr_5_O_12_, and did not release a sufficient amount of fluoride ions required to form a passive inner layer of MgF_2_.

The properties of coatings formed during PEO of AZ91D alloy using pulsed DC (*j* = 17 A/dm^2^, *f* = 500 Hz, *D* = 40%, *t* = 10 min) in electrolytes containing different amounts of NaF (3–9 g/L) to an acidic electrolyte composed of 5 g/L K_2_ZrF_6_, 6 g/L triethanolamine (TEA), 0.5 g/L NaH_2_PO_4_, and H_3_PO_4_ to adjust pH to 5.5–6.5 has been discussed in [85]. An increase in coating thickness was observed with increasing fluoride content in the electrolyte, from 12.7 µm without NaF to 50.1 µm with 9 g/L NaF. XPS analysis indicated the presence of MgO, ZrO_2_, and MgF_2_ phases in the coatings. The coating formed in the bath with 7 g/L NaF exhibited the highest fluorine content (25.07 at.%) and the most compact and unique self-sealing pore structure, which remained stable after 14 days of immersion in a solution of 3.5% NaCl. EIS analysis indicated that the improvement in corrosion resistance was mainly due to the presence of MgF_2_ in the barrier layer. The adhesion strength values, evaluated by scratch tests, indicated that the coating obtained with the 5 g/L NaF additive had the best mechanical properties. The important role of TEA was also observed, as it could chelate Mg^2+^ ions, inhibiting the corrosion of the magnesium substrate during the PEO process and moderating the pH of the baths.

The synergistic effects of NH_4_HF_2_ (4–8 g/L), K_2_ZrF_6_ (5–15 g/L), sodium phytate (Na_12_Phy) (8–16 g/L), and the treatment time (2.5–3.5 min) on the formation of conversion coatings on AZ31B alloy and their resistance to corrosion have been investigated using an orthogonal method [86]. The PEO process was carried out using pulsed DC with constant current control mode (*j* = 5 A/dm^2^, *f* = 2000 Hz, *D* = 35%). The addition of NH_4_HF_2_ to the bath was found to provide reactive F^−^ ions, which rapidly form MgF_2_. Na_12_Phy undergoes hydrolysis to produce Na_2_HPO_4_, which reacts with the substrate to form stable Mg_3_(PO_4_)_2_, which consequently seals the pores and forms a thicker coating. It was suggested that during PEO discharges, ZrF_6_^2−^ reacted with OH^−,^ producing F^−^ and Zr(OH)_5_^−^; the latter then led to ZrO_2_ and Mg_2_Zr_5_O_12_. However, an excess of K_2_ZrF_6_ caused discharge instability and the formation of cracks and pores in the coating. The presence of NH_4_HF_2_ in the electrolyte caused a significant increase in the amount of fluorine in the coating, while K_2_ZrF_6_ slightly decreased it, probably due to the competitive incorporation of zirconium. The Na_12_Phy additive decreased the content of fluorine and zirconium in the coating. The addition of NH_4_HF_2_ and Na_12_Phy to the bath inhibited the corrosion of the magnesium substrate. However, Na_12_Phy significantly improved the corrosion resistance of the coating, while NH_4_HF_2_ reduced it. K_2_ZrF_6_ was harmful to coating development and its high concentration increased the corrosion of the coating. However, the proper combination of all these ingredients ensured the formation of self-sealing coatings. The thickest coating, with the most compact structure and the best anti-corrosion properties, was that containing 4 g/L NH_4_HF_2_, 16 g/L Na_12_Phy, and 15 g/L K_2_ZrF_6_. A longer treatment time could increase the coating thickness, but in this case, it was the least important factor in corrosion resistance.

Due to the aggressive effect of acidic electrolytes on the magnesium substrate, the use of a two-step process, in which the first step was carried out in an alkaline environment, allowing the rapid formation of a passive layer on the magnesium, while the second involved the synthesis of the coating in an acidic solution containing K_2_ZrF_6_, has been a frequent solution [87,88].

The procedure has been applied for the PEO of AM50 alloy, in which a pulsed DC signal (*f* = 50 Hz) was used [87]. An initial “primary flash” oxidation step was performed in an alkaline environment (18 g/L NH_4_OH + 8 g/L Na_3_PO_4_, *j* = 3 A/dm^2^, *t* = unknown) and was followed by oxidation in an acidic bath (1 g/L NaH_2_PO_3_ + 3 g/L K_2_ZrF_6_, *U* = 420 V, *t* = 10 min). Furthermore, the coating obtained in this way was compared with that produced exclusively in an electrolytic solution of alkaline phosphate (*t* = 10 min). The coating obtained in the alkaline electrolyte was thinner (28 µm) and relatively compact, whereas the coating formed in the acidic electrolyte was thicker (40 µm) and had a large volume of fine pores, like a sponge. XRD measurements indicated the presence of MgO and an amorphous phase derived from phosphates in the coating produced in the alkaline bath. The presence of *t*-ZrO_2_, *m*-ZrO_2_, MgF_2_, and very little MgO was found in the coating obtained in the acidic electrolyte. EDS elemental line scan analysis of the coating cross-section indicated an increased amount of fluorine and a decreased amount of zirconium near the magnesium substrate. Corrosion resistance tests (PDP, EIS in 0.1 mol/L NaCl) showed that at the beginning of the corrosion test (0.5 h), the coating produced in the alkaline bath (without K_2_ZrF_6_) exhibited better barrier properties than the coating produced in the acidic bath (with K_2_ZrF_6_). However, increasing the exposure time (50 h) indicated that the coating produced in the presence of K_2_ZrF_6_ was characterized by greater stability and lower susceptibility to local corrosion.

Similar experiments have been carried out on AZ91 alloy using pulsed DC (*j* = 10 A/dm^2^, *f* = 500 Hz, *D* = 30%) [88]. In the first step, an alkaline phosphate solution (0.2 mol/L KOH + 0.03 mol/L Na_3_PO_4_·12H_2_O, pH = 12.2) was used and the oxidation process was carried out for 1 min. The second step was performed in an acidic electrolyte (0.02 mol/L H_3_PO_4_ + 0.02 mol/L K_2_ZrF_6_, pH = 2.7) for 10 min. XRD analysis indicated that the final coating was mainly composed of the Mg_2_Zr_5_O_12_ and ZrO_2_ phases; the amounts of MgO and MgF_2_ were minor. The SEM+EDS cross-sectional analysis indicated the presence of three layers (inner, intermediate, and outer) that differ in morphology and composition. ZrO_2_ was mainly located in the outer layer, while Mg_2_Zr_5_O_12_ was present in the intermediate layer. The amount of the MgF_2_ phase was negligible, which was attributed to the performance of the first oxidation step in the alkaline electrolyte. Two types of structures were observed on the surface of the resulting coating: a porous structure with large pores and a low-porosity structure that filled these pores. Based on the atomic ratios Mg:Zr and Zr:O, it was indicated that the structure with large pores was composed of Mg_2_Zr_5_O_12_, while the structure with fine pores consisted of ZrO_2_. It was proposed that in an acidic medium (pH ~ 3), K_2_ZrF_6_ formed ZrF_3_^+^ ions, which were converted to ZrO_2_ and Mg_2_Zr_5_O_12_ during the PEO process (Equations (11) and (12)). Furthermore, the presence of O^2−^ and F^−^ in the PEO process could lead to the formation of MgO and MgF_2_, respectively (Equations (13) and (8)).

ZrF_3_^+^ + 2O^2−^ → ZrO_2_ + 3F^−^ + e^−^ (11)

5ZrF_3_^+^ + 2Mg^2+^ + 14 O^2−^ → Mg_2_Zr_5_O_12_ + 15F^−^ +O_2_ + 4e^−^ (12)

Mg^2+^ + O^2−^ → MgO (13)

In subsequent research [89] the phase composition of the three layers of the coating produced from an acidic electrolyte containing K_2_ZrF_6_ has been investigated using HAADF-STEM with SAED analysis. It was found that the barrier layer consisted of nanocrystalline MgO and MgF_2_, the intermediate layer composed of ZrO_2_ polymorphs (monoclinic, tetragonal, and orthorhombic), and the outer layer contained MgO and *c*-ZrO_2_ (cubic).

A direct comparison has been presented between PEO coatings produced in an alkaline silicate bath (5 g/L NaOH + 15 g/L Na_2_SiO_3_) and in an acidic bath containing hexafluorozirconate (0.025 mol/L K_2_ZrF_6_ + H_3_PO_4_, pH = 2.5) [90]. Syntheses were performed on pure Mg using DC (*U* = 300 V, *t* = 3 min). In addition, a coating was produced in two steps, sequentially using the alkaline silicate electrolyte and the acidic zirconate electrolyte for 3 min each. XRD analysis indicated that the composition of the coating formed in the alkaline silicate electrolyte consisted of MgO, Mg(OH)_2_, and Mg_2_SiO_4_, whereas in an acidic environment (pH < 3) *t*-ZrO_2_, *m*-ZrO_2_, MgF_2_, KMgF_3_, Mg_2_Zr_5_O_12_, and the previously unreported ZrF_4_·H_2_O were formed (Equation (14)).

ZrF_3_^+^ + HF + H_2_O → ZrF_4_·H_2_O + H^+^ (14)

It was suggested that at high temperature and high pressure *t*-ZrO_2_ was formed, while rapid cooling of the electrolyte promoted its conversion to *m*-ZrO_2_. In the coating obtained in the two-step process, all the components present in both previous coatings were identified. Corrosion tests (PDP, EIS in HBSS) demonstrated better protective performance of the coating produced with the K_2_ZrF_6_ additive in the acidic electrolyte compared to that formed in the alkaline electrolyte (without K_2_ZrF_6_). However, the coating formed in the sequential processes exhibited the highest resistance.

The K_2_ZrF_6_ (10 g/L) additive to a conventional alkaline silicate bath (4 g/L KOH + 10 g/L Na_2_SiO_3_·9H_2_O, pH = 12.8) has been investigated [91] during the PEO of AZ91D alloy using pulsed DC (*U* = 300 V, *f* = 700 Hz, *D* = 30%, *t* = 5 min). The results obtained were compared with coatings produced in other zirconium-containing baths (10 g/L Zr(NO_3_)_4_·5H_2_O + 4 g/L KOH; pH = 2.3 and 10 g/L ZrOCl_2_·8H_2_O + 4 g/L KOH; pH = 12.2). Using a bath containing Zr(NO_3_)_4_, electrical discharges characteristic of the PEO process were not observed. In the presence of ZrOCl_2_ in a bath, spark discharges were visible for only 3 min, after which strong fluctuations in current values occurred. During synthesis in an alkaline electrolyte with K_2_ZrF_6_, discharges were visible until the end of the process. XRD analysis showed that ZrO_2_ was incorporated into all coatings. However, for Zr(NO_3_)_4_ in a bath, it was *m*-ZrO_2_, whereas in the other cases it was *t*-ZrO_2_, which has better strength properties. Furthermore, the presence of MgO, SiO_2_, MgF_2_, and Mg_2_SiO_4_ was detected in the K_2_ZrF_6_-derived coating, which is typical for this type of PEO coatings synthesized in alkaline baths. The coating synthesized with the addition of K_2_ZrF_6_ exhibited the best anti-corrosion properties (PDP in 3.5 wt.% NaCl), which were attributed to its greater thickness (13 µm compared to ~9 µm for the other two additives), compactness, and enriched phase composition.

In a comparative study [92], the properties of coatings produced with the addition of 0.02 mol/L (5.7 g/L) K_2_ZrF_6_ have been evaluated using single and two-step processes. The single-step process was carried out in an alkaline bath (0.05 mol/L KOH + 0.01 mol/L Na_3_PO_4_·12H_2_O + 0.02 mol/L K_2_ZrF_6_) for 10 min. In contrast, the two-step PEO process was performed initially in an alkaline electrolyte (0.02 mol/L KOH + 0.03 mol/L Na_3_PO_4_·12H_2_O) for 1 min, and then in an acidic bath (0.02 mol/L H_3_PO_4_ + 0.02 mol/L K_2_ZrF_6_) for 10 min. AZ31 alloy was used in the oxidation process with a pulsed DC (*j* = 10 A/dm^2^, *f* = 500 Hz, *D* = 30%). The coating produced in the single alkaline bath exhibited a morphology typical of that of PEO synthesis in alkaline electrolytes, characterized by porous pancake-like structures. Cross-sectional observations revealed two layers: an outer layer with huge micro-pores, severe cracks, and discharge channels, and a thin, compact inner layer. The coating produced in the two-step process (with an acidic K_2_ZrF_6_ bath) presented a porous pancake-shaped structure with sealed pores. The cross-section showed a triple-layer structure, comprising a porous outer layer, an intermediate layer with voids, and a compact inner layer. Both coatings had a thickness of approximately 23 µm. EDS surface analysis of the single-step process sample showed the presence of O, Mg, and Zr (51.6, 34.5, and 13.6 at.%, respectively). On the surface of the coating obtained from the two-step process, flat, porous structures were observed that were mainly composed of Zr (70.1 at.%) with small amounts of O, Mg, and F (20.1, 7.7, and 2.1 at.%), as well as structures that fill the pores with granular compounds consisting of O and Zr (63.3 and 33.4 at.%) and a small amount of Mg (3 at.%). Mechanisms for the formation of Mg_2_Zr_5_O_12_ were proposed in alkaline baths (Equation (15)) and acidic (Equation (12)) baths. Depending on the pH of the electrolyte, K_2_ZrF, existed in the forms of ZrF_3_^+^ or Zr(OH)_5_^−^ in acidic and alkaline baths, respectively. These ions are converted to Mg_2_Zr_5_O_12_ during the PEO process; additionally, the potential formation of ZrO_2_ in the acidic bath was indicated (Equation (11)).

5Zr(OH)_5_^−^ + 2Mg^2+^ + 2O^2−^ → Mg_2_Zr_5_O_12_ + 12H_2_O + OH^−^ + O_2_ + 4e^−^ (15)

XRD analysis indicated that the coating produced in the alkaline K_2_ZrF_6_ electrolyte was primarily composed of MgO. Minor signals from MgF_2_ and Mg_2_Zr_5_O_12_ were also detected, while ZrO_2_ was absent. In the case of the coating obtained in the acidic K_2_ZrF_6_ electrolyte, the Mg_2_Zr_5_O_12_ phase formed as the primary porous network, while ZrO_2_ acted as a filler within the pores. Corrosion tests (PDP and immersion test in 3.5 wt.% NaCl) demonstrated significantly superior anti-corrosion properties for the coating produced in the acidic electrolyte (two-step process), with a corrosion current density of 0.08 µA/cm^2^, compared to 1.56 µA/cm^2^ determined for the coating synthesized in the alkaline electrolyte (single-step process).

Modification of a conventional alkaline phosphate bath (2 g/L KOH + 12 g/L Na_3_PO_4_) by adding K_2_ZrF_6_ (2.5–10 g/L) has also been described [93]. The oxidation process was carried out on AZ31 alloy using a bipolar pulsed current (*j* = 8 A/dm^2^, *t* = 16 min). The presence of K_2_ZrF_6_ in the bath led to a systematic decrease in pH (from 10.5 for the K_2_ZrF_6_-free bath to 10.1, 9.6, and 8.7 after adding 2.5, 5, and 10 g/L, respectively) and an increase in conductivity (from 15.7 to 18.2, 22.5, and 29.3 mS/cm). A small addition of K_2_ZrF_6_ (2.5 g/L) resulted in a significant increase in the thickness of the coating, from 6.8 to 19.7 µm, compared to the base bath. However, a further increase in the concentration of K_2_ZrF_6_ to 5 and 10 g/L resulted in a decrease in the thickness of the coating, to 15.4 and 13.1 µm, respectively. The 2.5 g/L addition of K_2_ZrF_6_ also led to a reduction in roughness and pore size, probably due to the action of F^−^ ions derived from ZrF_6_^2−^. The increase in concentration to 5 and 10 g/L caused the formation of larger pores and the appearance of surface cracks. This was attributed to a decrease in the breakdown voltage and the occurrence of large destructive discharges during the final stage of the PEO process. XRD analysis showed that the K_2_ZrF_6_-modified coatings contained MgO, MgF_2_, and *t*-ZrO_2_. XPS measurements indicated that the coating produced in the base bath consisted primarily of Mg and O (62.2 and 36.7 at.%). The addition of K_2_ZrF_6_ to the electrolyte decreased the magnesium content and increased the oxygen content (to 45.9 and 47.3 at.%, respectively), for the concentration of 10 g/L, but also included a small amount of zirconium (3.1–3.4 at.%) and a trace amount of fluorine (0.3–0.8 at.%). The phosphorus content in the coatings was low and increased only slightly after the addition of K_2_ZrF_6_ (0.9–1.8 at.%). Corrosion studies (PDP, EIS, and immersion tests in 3.5 wt.% NaCl) demonstrated that the K_2_ZrF_6_ additive positively affects anti-corrosion properties. The best barrier performance was exhibited by the coating produced with the 2.5 g/L K_2_ZrF_6_ addition.

Optimization of an alkaline silicate bath supplemented with K_2_ZrF_6_ (5–15 g/L) has been performed for the PEO of AZ91D alloy using an AC (*U* = 300 V, *f* = 480 Hz, *D* = 30%, *t* = 10 min) [94]. The base electrolyte (20 g/L Na_2_SiO_3_·9H_2_O + 5 g/L NaF) was enriched with components typically found in acidic hexafluorozirconate baths (8 g/L NH_4_H_2_PO_4_ and 5 g/L trisodium citrate). It is worth noting the absence of hydroxides (KOH/NaOH) in the alkaline baths used in this study. The thicknesses of the resulting coatings ranged from 5 to 8 µm. The coating formed only with silicate and sodium fluoride (base bath) was porous and had numerous crater-shaped discharge channels. The introduction of dihydrogen phosphate and citrate led to the formation of a more compact structure, with slightly improved anti-corrosion properties (a decrease in the corrosion current density from 7.48 to 6.57 µA/cm^2^). In addition to Mg, Al, O, Si, and F, P was also incorporated into the coating. When K_2_ZrF_6_ was subsequently introduced into this modified electrolyte, the porosity of the coating was further reduced and zirconium was integrated into its structure. A continuous decrease in *j*_corr_ values was observed with the increase in K_2_ZrF_6_ concentration, giving 0.98, 0.67, and 0.40 µA/cm^2^ for 5, 10, and 15 g/L, respectively. The cross-section of the coating showed a three-layer structure. EDS analysis indicated that the inner layer consisted mainly of Mg, Al, and O, the intermediate layer was composed of F, P, and Zr, and the outer layer was dominated by Mg, Zr, and P. XRD analysis showed that the coating without the K_2_ZrF_6_ additive was mainly composed of MgO, MgSiO_3_, and MgF_2_. The addition of K_2_ZrF_6_ resulted in further incorporation of *t*-ZrO_2_. The combined results of EDS and XRD suggest that the inner layer was made up mainly of MgO with small amounts of Al_2_O_3_, while the intermediate layer was dominated by MgF_2_ and ZrO_2_. The surface analysis of the outer layer indicated that the areas surrounding the pores contained MgF_2_, ZrO_2_, MgO and magnesium-related phosphates, while the pores themselves were filled with MgF_2_. It was stated that PEO was a complex process that involved electrochemical, thermochemical, and plasma reactions. Even before the beginning of plasma discharges, certain anions such as SiO_3_^2−^, PO_4_^3−^, ZrF_6_^2−^, OH^−^, and F^−^ migrated toward the magnesium substrate. There, they reacted with Mg^2+^ to form a pre-coating, which can cause the current to pass unevenly through the surface. Once sufficient voltage was reached, electrically conductive channels were formed. High-temperature discharges triggered reactions among anions located in the vicinity of these channels. Furthermore, electrical discharges accelerated the hydrolysis of ZrF_6_^2−^, leading to an increasing amount of F^−^ and ZrO_2_ within the discharge channel (Equation (16)).

ZrF_6_^2−^ + 4OH^−^ → ZrO_2_ + 6F^−^ +2H_2_O (16)

The molten material ejected from the discharge channels solidified around the pores, resulting in the formation of mountain-like features. Changes in the layer composition of the K_2_ZrF_6_-modified coating were attributed to differences in the melting and boiling points of its components: ZrO_2_ (2680 and 4300 °C), MgO (2852 and 3600 °C), MgF_2_ (1890 and 2239 °C). Because ZrO_2_ has an extremely high melting and boiling point, it did not melt or evaporate inside the discharge channel, unlike the other phases. Instead, it remained solid and accumulated within the discharge pores, while molten MgF_2_ and MgO flowed and solidified around it. This led to progressive sealing of the discharge channels and the formation of a dense intermediate layer rich in MgF_2_ and ZrO_2_ beneath the porous outer layer. The interiors of the external pores were filled with MgF_2_ due to its relatively low melting point.

A sequence of studies [95,96,97,98] has been dedicated to the optimization of synthesis parameters during the production of coatings on magnesium alloys produced in an alkaline silicate bath (3.5 g/L NaOH + 12 g/L Na_2_SiO_3_·10 H_2_O + 0.3 g/L Na_2_SiF_6_) and also with the addition of K_2_ZrF_6_. The PEO process was conducted using a bipolar hybrid voltage signal (DC 260 V + AC 200 V).

A series of syntheses has been performed on AZ91D alloy using a two-step process, in which K_2_ZrF_6_ (6 g/L) was introduced only in the second bath [95]. The influence of the duration of the first stage (5, 15, or 30 min) on the properties of the resulting coatings was investigated, while the second stage always lasted 10 min. The two-step coatings consisted of MgF_2_, ZrO_2_, MgO, and Mg_2_SiO_4_. The mixed mechanism for the incorporation of ZrO_2_ into the coating was proposed, which involves the hydrolysis of K_2_ZrF_6_ to colloidal Zr(OH)_4_ in an alkaline environment (Equation (17)) and the direct reaction between ZrF_6_^2−^ and OH^−^ ions, which occurs only during the PEO process (Equation (16)).

ZrF_6_^2−^ + 4OH^−^ → Zr(OH)_4_ + 6F^−^ (17)

The value of the isoelectric point of Zr(OH)_4_ is 6.8; therefore, in an alkaline environment, it exists in electronegative forms, such as [ZrO(OH)_3_]^−^, ZrO(OH)_4_]^−^, [Zr(OH)_5_]^−^. Due to this negative charge and under the influence of the applied voltage, these species can migrate toward the anode and react to produce ZrO_2_. The coatings oxidized for 15 min during the first stage showed the best anti-corrosion properties and also exhibited the lowest porosity and the highest hardness.

In subsequent research [96] a single-step PEO of AZ31B alloy has been performed using an electrolyte with a fixed composition of 3 g/L NaOH, 12 g/L Na_2_SiO_3_·10 H_2_O, 0.3 g/L Na_2_SiF_6_, and 6 g/L K_2_ZrF_6_ at different times of the process, from 5 to 20 min. It was observed that increasing the synthesis time from 5 to 15 min resulted in a linear growth of the coating thickness (from ~65 to ~80 µm), whereas its sudden increase took place at 20 min (~130 µm). The coatings produced at 10 and 15 min were characterized by the lowest surface porosity (~1.5% and ~2.5%, respectively). All coatings exhibited a triple-layer structure consisting of a thin barrier layer adjacent to the substrate, a thick but porous intermediate layer, and a dense top layer. Extending the oxidation time to 20 min led to an increase in pore size within the intermediate layer. Corrosion tests (PDP in 3.5 wt.% NaCl) indicated that the coating synthesized for 15 min possessed the best barrier properties. Meanwhile, hardness measurements showed a steady increase with increasing synthesis time (950–1250 HV for 5–20 min). XRD analysis defined the phase composition of the coatings as MgF_2_, ZrO_2_, Mg_2_SiO_4_, and MgO. The potential formation of ZrSi_2_ was suggested, a finding that had not been previously reported.

Optimization of K_2_ZrF_6_ concentration (ranging from 2 to 8 g/L) in an alkaline silicate bath used in the second step of the PEO process of AZ91D alloy has also been reported [97]. A concentration of 6 g/L was found to be optimal, providing coatings with the highest thickness, lowest porosity, highest hardness, and superior anticorrosive performance. An excessive increase in the concentration of K_2_ZrF_6_ led to the deterioration of the inner barrier layer due to the increase in acidity induced by ZrF_6_^2−^ ions. Moreover, the formation of colloidal particles, resulting from the finite solubility of the salt, could lead to discharge instability during the process.

Finally, the incorporation of ZrO_2_ nanoparticles (200 nm) present in the electrolyte into the coatings formed on AZ91D alloy has been described [98]. The two-stage PEO process (the first 15 min, the second 15 or 30 min) was applied. In the second step, an alkaline silicate bath with 6 g/L K_2_ZrF_6_ and ZrO_2_ nanoparticles at concentrations of 2 to 8 g/L was used. The increase in the ZrO_2_ content in the electrolyte was found to result in a higher amount of incorporated Zr. Morphological changes in the surface were observed, shifting from a typical pancake-type structure to a cluster-type structure. However, an excessive concentration (6–8 g/L) of nanoparticles caused their agglomeration near the discharge channels, which consequently resulted in process instability and a non-uniform distribution of zirconium on the coating surface. The complex nature of the incorporation of ZrO_2_ nanoparticles in the PEO process was indicated, involving various simultaneous processes such as electrophoretic forces, physical mixing of particles due to plasma shock waves, gas evolution, and internal fluctuations of molten MgO. Only a small addition of ZrO_2_ nanoparticles (2–4 g/L) effectively sealed the pores without significantly altering the overall appearance of the coating. Cross-sectional images indicated that only the coating containing 2 g/L ZrO_2_ and oxidized for 15 min was free of defects in the inner layer. Consequently, this coating exhibited the best anti-corrosion properties (PDP, EIS in 3.5 wt.% NaCl). Extension of the oxidation time caused an increase in the coating thickness, but also led to its structural damage.

Similarly, the effect of the presence of 2 g/L of ZrO_2_ nanoparticles (200 nm) in an alkaline phosphate bath (3 g/L NaOH + 6 g/L (NaPO_3_)_6_ + 10 mL/L TEA) with the addition of K_2_ZrF_6_ has been reported [99]. AZ91D alloy was subjected to the PEO process using a pulsed DC (*j* = 3 A/dm^2^, *f* = 300 Hz, *D* = 25%, *t* = 15 min). Initially, the concentration of K_2_ZrF_6_ was optimized in the range of 4 to 16 g/L. Its introduction into the electrolyte led to the incorporation of Zr and F into the resulting coating, and their contents increased from 4.3 to 9.2 at.% and from 2.4 to 6.9 at.%, respectively. XRD analysis confirmed the presence of crystalline MgO and, in the presence of K_2_ZrF_6_, the formation of Mg_2_Zr_5_O_12_. It was suggested that K_2_ZrF_6_ in an alkaline environment formed colloidal ZrO_2_·nH_2_O particles, which then adsorbed on the magnesium surface and reacted during the PEO process, filling the pores (self-sealing effect). The thickness of the coating increased from 23 to 35 µm (for 0–10 g/L K_2_ZrF_6_), but the diameter of the external pores decreased from 40 to 10 µm. When the K_2_ZrF_6_ content exceeded 10 g/L, the size and quantity of holes in the coatings increased, the thickness decreased (33 µm for 16 g/L K_2_ZrF_6_), and the self-sealing effect was reduced. This behavior was attributed to the influence of a higher concentration of F^−^ ions, which were also released during the hydrolysis of K_2_ZrF_6_. EIS analysis indicated that the coating with 10 g/L K_2_ZrF_6_ exhibited the best anticorrosive performance. The presence of ZrO_2_ nanoparticles in the optimized electrolyte caused their partial entry into the pores and their reaction with MgO at plasma temperatures to form Mg_2_Zr_5_O_12_. This resulted in an increase in thickness (from 35 to 45 µm), enhanced compactness, and improved anti-corrosion properties of the coating obtained.

The development and modification of alkaline hexafluorozirconate electrolytes remain a subject of intense scientific interest. Beyond ZrO_2_ incorporation, various studies have shown the beneficial effects of the use of alternative nanoparticles, such as titanium carbide (TiC), niobium carbide (NbC) [100], or graphite [101]. The efforts to optimize electrolyte composition are still ongoing, for example, by replacing K_2_ZrF_6_ with NH_4_ZrF_6_ [102,103], or by employing complex base electrolytes such as aluminate-phosphate [104] or silicate-aluminate [105].

#### 4.2.2. TiF_6_^2−^

Hexafluorotitanate is another complex fluoride ion utilized in the PEO of magnesium alloys. The effect of adding K_2_TiF_6_ (10 g/L) to an alkaline phosphate bath consisting of 3 g/L NaOH, 5 g/L Na_5_P_3_O_10_, and 1 g/L (NaPO_3_)_6_ has been investigated in the PEO of Mg–8.5Li–1Al alloy using pulsed DC (*j* = 5 A/dm^2^, *f* = 1000 Hz, *D* = 15%, *t* = 15 min) [106]. The coating produced in the base electrolyte (without K_2_TiF_6_) exhibited a typical structure for PEO. The surface contained numerous open pores, and in the cross-section, two distinct layers included a thick, porous outer layer and a thin, dense inner layer were clearly visible. This coating was composed of O, Mg, and P (42.2, 37.6, and 16.2 at.%, respectively), and the distribution of the elements throughout the coating was uniform. The introduction of K_2_TiF_6_ into the bath led to the incorporation of 12.0 at.% Ti and 8.3 at.% F into the coating, reducing the contents of O, Mg, and P to 39.0, 28.4, and 8.2 at.%, respectively. Significant modifications in the coating morphology were also observed. Regions enriched in O and Ti (44.8 and 21.9 at.%, respectively) were observed on the coating surface, and some of the pores were sealed by a structure with a high fluorine content (29.1 at.%). The coating obtained in the presence of K_2_TiF_6_ was thicker than the one produced without this additive, and its cross section showed a unique sandwich-like structure containing a dense inner layer, a dense outer layer, and an intermediate layer with some sealed or semi-sealed pores. XRD analysis indicated that the base coating was composed of MgO and non-crystalline phosphates. The modified coating consisted of MgF_2_, Ti_3_O_5_, Ti_6_O_11_, and MgO. Cross-sectional compositional analysis indicated that the outer layer was rich in titanium, whereas the inner layer and pore fillings exhibited an increased amount of fluorine. Corrosion tests (PDP, EIS in 3.5 wt.% NaCl) confirmed the superior anticorrosive performance of the modified coating. The formation of a white emulsion after the addition of K_2_TiF_6_ to the bath was indicated, which was attributed to the partial hydrolysis of TiF_6_^2−^ ions in the alkaline environment (Equation (18)).

TiF_6_^2−^ + 4OH^−^ → TiO_2_ + 6F^−^ + 2H_2_O (18)

However, while discussing the mechanism of coating formation, the occurrence of direct thermochemical reactions of the unhydrolyzed TiF_6_^2−^ ions during the PEO process, resulting in the formation of MgO, TiO_x_ (Ti_3_O_5_, Ti_6_O_11_), MgF_2_, and F^−^, was considered. It was emphasized that MgF_2_ exhibits a higher product-to-metal ratio (PMR: *V*_product_/*V*_metal_) than MgO (1.42 and 0.81, respectively), which favors the sealing of the coating. Moreover, the lower melting and boiling temperatures of MgF_2_ compared to MgO indicate that MgF_2_ exhibits superior flow properties, allowing it to preferentially fill the pores.

A direct comparison of the properties of the coating produced in the presence of K_2_TiF_6_ with other additives commonly used in alkaline PEO baths for magnesium alloys has been reported [107]. An alkaline silicate solution (4 g/L NaOH + 10 g/L Na_2_SiO_3_·9H_2_O) served as the base electrolyte and additions of K_2_TiF_6_, Na_3_PO_4_·10H_2_O, NaAlO_2_, Na_2_B_4_O_7_·10H_2_O, and urea were applied, all at a concentration of 2 g/L. AZ31 alloy was subjected to the PEO process with a pulsed DC signal (*j* = 6 A/dm^2^, *f* = 50 Hz, *D* = 50%, *t* = 10 min). The coating produced using the K_2_TiF_6_ additive was found to have a low external pore density; the average pore size and the percentage porosity area decreased from 22.9 to 7.4 µm and from 4.8 to 2.3%, respectively, compared to the base electrolyte. The only additive that further reduced porosity was Na_2_B_4_O_7_. The coating obtained in the presence of K_2_TiF_6_ also showed the highest thickness (50 µm). XRD analysis indicated the presence of MgO and Mg_2_SiO_4_ phases in all the coatings investigated and the addition of K_2_TiF_6_ to the electrolyte led to the formation of the MgF_2_ phase. Corrosion tests (PDP, EIS in 3.5 wt.% NaCl) showed an improvement in anti-corrosion properties relative to the base coating when K_2_TiF_6_ was used as an additive. Among the other additives, only the coating produced with Na_2_B_4_O_7_ addition demonstrated superior barrier performance, which the authors confirmed in their subsequent study [108].

An alkaline glycerophosphate bath (6 g/L NaOH + 6 g/L calcium glycerophosphate + 4 g/L K_2_TiF_6_) has been used to produce a coating on AZ31 alloy with monopolar pulsed DC (*U* = 400 V, *f* = 1000 Hz, *D* = 10%, *t* = 10 min) [109]. The coating obtained was approximately 5 µm thick and contained Mg, F, O, Ti, P, and Ca. XRD analysis showed the presence of MgO, TiO_2_, and CaTiO_3_ phases, whereas no crystalline phases containing phosphorus or fluorine were detected. The TiF_6_^2−^ ion was readily hydrolyzed in an aqueous environment to form Ti(OH)_4_, while Ca^2+^ ions released from calcium glycerophosphate reacted with OH^−^ to form Ca(OH)_2_. The formation of the CaTiO_3_ phase during the PEO process was explained by assuming the dehydration of these hydroxides to produce TiO_2_ and CaO, respectively, followed by their plasma-induced high-temperature fusion. Electrochemical tests (PDP in SBF) showed a substantial improvement in the corrosion resistance of the coating compared to that of the uncoated magnesium alloy. Surface analysis after 7 days of immersion in SBF showed that the entire surface was covered with spherical particles, which exhibit a high ability to form apatite, indicating potential usefulness in implantology applications.

A comprehensive evaluation of the influence of the addition of 12 g/L K_2_TiF_6_ to an alkaline phosphate bath (3 g/L NaOH + 6 g/L (NaPO_3_)_6_ + 10 mL/L TEA) on the properties of conversion coatings formed on AZ91D alloy has been presented in [110]. The process was carried out using pulsed DC (*j* = 3 A/dm^2^, *f* = 200 Hz, *D* = 15%, *t* = 15 min). The introduction of K_2_TiF_6_ increased the electrolyte conductivity from 13.0 to 16.5 mS/cm, resulting in a reduction in the breakdown and final voltages from 220 to 180 V and from 545 to 524 V, respectively, compared to the base bath. Furthermore, the addition of K_2_TiF_6_ drastically reduced the pH of the electrolyte from 12.4 to 6.8 and caused the appearance of a milky white turbidity of the electrolyte, indicating that hydrolysis reactions occur in the Ti-containing electrolyte. Dynamic light scattering showed the presence of large particles with average diameters of approximately 370 nm and 2990 nm in the modified bath, in contrast to the 2 nm and 103 nm observed for the base electrolyte. The base electrolyte was not stable. After 1 day of storage, the coating produced was nonuniform and only minor sparking occurred during the PEO process. By comparison, the K_2_TiF_6_-modified electrolyte exhibited remarkable stability for more than 2 months (despite initial hydrolysis), allowing reproducible syntheses. In the course of the PEO process particles formed during hydrolysis of K_2_TiF_6_ such as TiO_2_ molecules and TiF_6_^2−^, F^−^, OH^−^, and P_6_O_18_^6−^ anions are drawn into the discharge channels by migration and/or adsorption, reacting with the magnesium substrate. It was shown that the addition of 12 g/L K_2_TiF_6_ nearly doubled the coating thickness (from 20.3 to 39.8 µm), while reducing the number and size of surface pores. The coating formed in the base electrolyte was composed of MgO and amorphous phosphates, whereas the presence of K_2_TiF_6_ in the electrolyte led to the formation of MgF_2_, Mg_2_PO_4_F, Mg_2_TiO_4_, and TiO_2_ (anatase). The improved anticorrosive performance of the modified coating was attributed to the formation of the TiO_2_ and Mg_2_TiO_4_ phases, the last under the conditions of the PEO process (Equation (19)).

TiO_2_ + 2MgO → Mg_2_TiO_4_ (19)

Furthermore, the layers formed in the presence of K_2_TiF_6_ exhibited photocatalytic activity due to the presence of TiO_2_ on their surfaces, making them promising for applications such as solar cells, photocatalytic waste degradation, and antimicrobial coatings.

The PEO of AM60 alloy in an alkaline phosphate bath (1–4 g/L NaOH + 4–8 g/L (NaPO_3_)_6_ + 0.5–3 g/L Na_5_P_3_O_10_ + 8–10 g/L K_2_TiF_6_) has also been investigated [111]. The bath components were dissolved separately in deionized water and subsequently mixed together. After the mixing process, white sol–gel precipitates were observed. Initially, the pH was 11, but it gradually decreased and stabilized at 6 as a result of a spontaneous decomposition reaction in the electrolyte solution, which takes several hours to complete. XRD analysis identified the white precipitate as amorphous TiO_2_. This indicates that the observed changes in the electrolyte were caused by the hydrolysis of TiF_6_^2−^ ions (Equation (20)).

TiF_6_^2−^ + 2OH^−^ → TiO_2_ + 6F^−^ + 2H^+^ (20)

PEO syntheses were performed using pulsed DC (*f* = 1000 Hz, *D* = 30%). The process was initially operated at constant current (*j* = 5 A/dm^2^) to reach voltages of 60, 100, 200, 300, and 420 V and then kept at 420 V for some minutes. For the sample anodized to 60 V, no visible coating was observed and only a few bright spots were detected on the alloy surface. However, EDS analysis indicated that fluorine (2.5 wt.%) was incorporated into the layer at this stage, which was attributed to the formation of an ultra-thin MgF_2_ layer. No other electrolyte-derived components were detected at this voltage. At 100 V, a loose and non-uniform layer was identified, which already incorporated all elements introduced from the bath (F, Na, K, P, and Ti). After reaching a voltage of 200 V, the coating exhibited a porous morphology characteristic of the PEO process, reaching a thickness of 2 µm. At 300 V, the thickness increased to 6 µm, and a partial filling of some pores occurred. When the voltage reached 420 V, the coating thickness increased to 20 µm, and most of the pores were sealed. Further oxidation (holding 420 V for 2 min) did not lead to a significant increase in the coating thickness. However, it resulted in reduced pore density, increased pore diameter, and ultimately complete pore filling. Specifically, the diameter of the pores expanded from 1 to 20 µm between the 200 V and 420 V stages. Elemental analysis of the pore fillings indicated that they were predominantly composed of Mg (31.1 wt.%), F (26.3 wt.%), and O (25.4 wt.%). In contrast, the outer structure of these pores consisted of O (38.4 wt.%), Mg (23.6 wt.%), Ti (13.9 wt.%), P (10.3 wt.%), F (9.8 wt.%), and Na (9.2 wt.%). Cross-sectional analysis of the coatings showed an enrichment of fluorine near the magnesium substrate, whereas an increased concentration of titanium was found in the outer layer of the coating. XRD measurements indicated the presence of MgO, MgF_2_, Mg_2_TiO_4_, Ti_3_O_5_, and Na_2_MgP_2_O_7_ phases. The pores were filled with a structure rich in MgF_2_. The blue color of the coating was associated with the presence of the Ti_3_O_5_ phase, which forms through the decomposition of TiO_2_ at plasma temperatures (Equation (21)).

6TiO_2_ → 2Ti_3_O_5_ + O_2_ (21)

A comprehensive optimization of the PEO of AZ91D alloy has been investigated [112] using monopolar pulsed DC (*j* = 3–10 A/dm^2^, *f* = 200 Hz, *D* = 15%, *t* = 15 min) in alkaline phosphate electrolytes composed of K_2_TiF_6_ (2–12 g/L), NaOH (1–5 g/L), (NaPO_3_)_6_ (2–14 g/L), and 10 mL/L TEA. Initially, the concentration of K_2_TiF_6_ was optimized for constant values of the other parameters 3 g/L NaOH, 6 g/L (NaPO_3_)_6_, and *j* = 3 A/dm^2^. The observed decrease in pH (approximately from 12.5 to 6.5) with the increase in K_2_TiF_6_ concentration was related to alkaline hydrolysis of TiF_6_^2−^ to Ti(OH)_4_ sol particles. The conductivity initially decreased to K_2_TiF_6_ concentration equal to 4 g/L, which was attributed to the consumption of OH^−^ ions during hydrolysis, combined with the adsorption of Na^+^ and K^+^ ions on the sol particles formed. The subsequent increase in conductivity for K_2_TiF_6_ concentrations (4–12 g/L) was associated with the release of free K^+^ and F^−^ ions. The introduction of a small amount of K_2_TiF_6_ (2 g/L) significantly promoted coating growth, increasing its thickness from 20 to 32 µm. For the further increase in additive concentration, this effect became considerably less pronounced, with the coating reaching a thickness of 39.8 µm at 12 g/L. The increase in K_2_TiF_6_ concentration up to 8 g/L led to a reduction in the diameter of the pores. For higher concentrations, the pores became larger and some large molten oxide particles were observed on the coating surface. The highest titanium content in the coating was found to be 13.2 at.% for 8 g/L K_2_TiF_6_ in the electrolyte. It was explained by the assumption that TiO_2_ colloidal particles were drawn into discharge channels during plasma oxidation. The increase in K_2_TiF_6_ concentration caused an increase in the diameter of colloidal agglomerates, subsequently inhibiting their movement into the discharge channels. In contrast, the amount of fluorine incorporated increased steadily, reaching 9.6 at.% for 12 g/L of the K_2_TiF_6_ additive. XRD analysis indicated that the coating produced without K_2_TiF_6_ was composed of MgO, MgAl_2_O_4_, and some phases of amorphous phosphorus. The coatings obtained with the addition of K_2_TiF_6_ consisted of Mg_2_TiO_4_, Mg_2_PO_4_F, and MgF_2_. For concentrations of 8 g/L or higher, a TiO_2_ signal was also detected. EIS measurements showed that the coating prepared with 8 g/L of K_2_TiF_6_ exhibited the best anticorrosive properties in 3.5 wt.% NaCl. Optimization of the concentration of (NaPO_3_)_6_ in an electrolyte composed of 3 g/L NaOH, 8 g/L K_2_TiF_6_, and 10 mL/L TEA was shown that (NaPO_3_)_6_ acted as a surfactant, adsorbing on the surface of TiO_2_ colloids to prevent their agglomeration. A white precipitate was observed in the electrolyte with concentrations of (NaPO_3_)_6_ lower than 2.0 g/L, indicating that colloidal TiO_2_ particles could not exist stably in these solutions. Moreover, a coating with loose white attachments was formed in these electrolytes. The optimal anti-corrosion performance was found for the bath containing 8 g/L (NaPO_3_)_6_. The NaOH concentration was also optimized using a solution of 8 g/L K_2_TiF_6_, 8g/L (NaPO_3_)_6_, and 10 mL/L TEA. The sample produced with 3 g/L NaOH exhibited the best corrosion resistance. Among the current densities applied in the PEO process (3, 6, and 10 A/dm^2^), the coating produced at 6 A/dm^2^ showed the highest impedance. An increase in current density led to a simultaneous growth in both the coating thickness (43, 52, and 70 µm) and the pore diameter (25, 35, and 40 µm). The coating obtained at 10 A/dm^2^ exhibited larger pores and cracks and therefore poorer protective properties.

Optimization of K_2_TiF_6_ concentration in a range of 2–10 g/L has also been performed [113] for PEO process of AZ80 alloy using pulsed DC (*U* = 400 V, *f* = 400 Hz, *D* = 4%, *t* = 15 min). An electrolyte composed of an alkaline silicate bath (12 g/L Na_2_SiO_3_ + 5 g/L NaF + NaOH adjusted to pH 12) and 3 g/L hydroxyapatite nanoparticles (Ca_10_(PO_4_)_6_(OH)_2_, HAp) with 10 mL/L ethylene glycol. The presence of K_2_TiF_6_ in the electrolyte led to an increase in the coating thickness, ranging from 10 to 20 µm for 0–5 g/L of the additive. However, a further increase in its concentration resulted in only a slight thickness change, reaching 21 µm at 10 g/L K_2_TiF_6_. Increasing the K_2_TiF_6_ content reduced both the size and number of pores. Significant surface changes occurred at 10 g/L, where some pores were sealed with bath constituents and others were covered by the additional layer. The coating composition contained Mg, O, F, P, Ca, Si, and Ti (after the addition of K_2_TiF_6_), which was caused by the numerous components present in the bath. With an increasing amount of K_2_TiF_6_, a systematic growth in the content of fluorine (from 12.9 to 18.01 at.%) and titanium (from 1.21 to 2.57 at.%) was observed in the coating. However, a high amount of fluorine (7.43 at.%) was already found in the coating produced in the base bath containing only NaF. XRD analysis showed that all the coatings formed were made up of MgF_2_, Mg_2_SiO_4_, HAp, and MgO. The introduction of K_2_TiF_6_ induced the appearance of the Ti_3_O_5_ phase. EIS measurements during the long-term corrosion tests performed (SBF, 280 h) indicated that the coating produced with the addition of 10 g/L K_2_TiF_6_ exhibited the best anti-corrosion properties. After 280 h of immersion in SBF, the coating exhibited a Ca/P molar ratio equal to 1.77, which is close to the value of 1.67 appropriate for the osteogenic activity of the materials, which indicated its good biocompatibility and the possibility of improving its biodegradation in a biological environment.

K_2_TiF_6_, 3–9 g/L, has also been used [114] as an additive to an alkaline silicate electrolyte composed of 8 g/L KOH and 6 g/L liquid-glass sodium silicate ((Na_2_O)_x_·SiO_2_, 37 wt.%). The reagents were dissolved separately and then mixed together. The PEO process on AZ31 alloy was carried out using a pulsed DC (*U* = 440V, *f* = 1000 Hz, *D* = 10%, *t* = 10 min). The introduction of K_2_TiF_6_ into the electrolyte caused a systematic decrease in pH from 12.7 to 11.2 for the range of concentrations 0–9 g/L. The conductivity first decreased from 18.1 mS/cm without K_2_TiF_6_ to 15.9 mS/cm at 5 g/L K_2_TiF_6_, and subsequently increased to 16.9 mS/cm at 9 g/L. This behavior was previously described [112]. It was indicated that regardless of the hydrolysis of TiF_6_^2−^ at pH ≥ 6 to form colloidal TiO_2_ (Equation (20)), the precipitation of colloidal TiO_2_ particles could be prevented by careful control of the order of addition of the electrolyte components. The introduction of 3 g/L K_2_TiF_6_ resulted in a substantial increase in the thickness of the coating, from 10.2 to 19.1 µm. Further increases in additive concentration had a less pronounced effect, with the thickness reaching 24.8 µm at 9 g/L K_2_TiF_6_. The porosity of the coating initially decreased from 26% to 15% for the 0–5 g/L range, but then increased to 22% at 9 g/L. The achievement of the lowest porosity for the coating modified with 5 g/L K_2_TiF_6_ was directly correlated with the minimum oxidation current recorded under constant voltage. With an increasing amount of K_2_TiF_6_ (3–9 g/L), an increase in the content of fluorine (from ~1.5 to ~5.8 at.%) and titanium (from ~2.8 to ~4.8 at.%) was observed. The silicon content initially decreased from ~24 to ~14 at.% for the 0–5 g/L K_2_TiF_6_ range and then remained at a similar level (~14 at.%). In the coating formed in the base electrolyte, the presence of crystalline phases of MgO, Mg_2_SiO_4_, and MgSiO_3_ was identified. After the introduction of K_2_TiF_6_, the presence of MgF_2_ and TiO_2_ (anatase) was also detected. Elemental mapping images (SEM+EDS) showed fluorine enrichment within the barrier layer and uniform incorporation of titanium. The coating produced with the addition of 5 g/L K_2_TiF_6_ exhibited the best anti-corrosion performance, which was confirmed by EIS tests after exposure in 3.5 wt.% NaCl for 7 days. The mechanism of coating growth was investigated by analyzing the initial stages of synthesis (30, 60, and 150 s) in electrolyte with 5 g/L K_2_TiF_6_. During the first stage (30 s), local defects resulting from substrate dissolution and localized oxygen-rich precipitates were visible on the metal surface. At this point, only oxygen and fluorine were incorporated into the substrate structure, suggesting the formation of a thin layer composed of MgO and MgF_2_. Extending the oxidation time to 60 s enabled to reach the preset potential of 440 V. Consequently, a porous coating was formed that also contained silicon and titanium; however, a decrease in fluorine content was observed. Furthermore, some of the pores were sealed by a titanium-rich structure. It was suggested that only small TiO_2_ particles are drawn into the outer coating layer, whereas particles larger than the diameter of the discharge channels were returned to the solution after the discharge was shut down. The PEO process became more intense at 150 s, leading to the formation of larger pores on the coating surface. As a result, SiO_3_^2−^ ions and larger colloidal TiO_2_ particles accumulated inside the pores, where they melted due to the high temperature caused by plasma discharges.

Research focusing on optimizing the conditions for coating formation on magnesium alloys by the PEO process using electrolytes containing K_2_TiF_6_ is still ongoing. These efforts emphasize both the further optimization of the electrolyte composition [115,116] and the modification of electrical parameters, such as employing a bipolar current signal to achieve a soft sparking regime [117].

A direct comparison of the effects of additions of K_2_TiF_6_ and K_2_ZrF_6_ (5 g/L) to the neutral phosphate bath (5 g/L NaH_2_PO_4_ + 15 g/L KF; pH = 6.5) on the properties of PEO coatings formed under pulsed DC (*j* = 2 A/dm^2^, *f* = 500 Hz, *D* = 30%, *t* = 5, 10, and 30 min) on AZ91D alloy has also been described [118]. The introduction of these additives into the base bath decreased the anodizing voltage, which reduced the spark size and consequently led to a reduction in the coating porosity. The pores of all the coatings obtained were partially sealed with MgF_2_. The application of a neutral pH resulted in a lower amount of oxygen being incorporated into the coatings compared to the traditional layers produced in alkaline electrolytes. However, large amounts of fluorine incorporated (57–59 at.%) were recorded. Mg, Al, K, and P were also detected in all coatings. The introduction of K_2_TiF_6_ and K_2_ZrF_6_ additives led to the additional incorporation of Ti and Zr, respectively. Cross-sectional composition analysis indicated that fluorine was uniformly distributed throughout the volume of all coatings. In contrast, titanium and zirconium accumulated near the metal/coating interface. The thicknesses of the coatings obtained in the presence of K_2_TiF_6_ and K_2_ZrF_6_ increased with oxidation time and their values were approximately the same for both additives in the same time intervals. XRD analysis showed that the coatings were mainly composed of MgF_2_ and KMgF_3_, together with a smaller amount of MgO. When additives were used, the coatings also contained TiO_2_ or ZrO_2_. Investigations of anti-corrosion performance (PDP and EIS in a 3.5 wt.% NaCl) and mechanical properties (hardness and adhesion force) demonstrated their enhancement in the presence of both additives in the bath. Slightly better mechanical properties were obtained with the addition of K_2_ZrF_6_, whereas superior corrosion resistance was observed for the coating modified with K_2_TiF_6_.

#### 4.2.3. SiF_6_^2−^

Several studies have reported the influence of other complex fluorine salts used as additives in electrolyte for PEO processes on the properties of conversion coatings on magnesium alloys. In a series of articles, the role of Na_2_SiF_6_ was described [119,120,121,122].

In the study [119], the PEO process of AZ31B alloy has been carried out using a hybrid voltage configuration (260 V DC + 200 V AC) for 30 min. An alkaline silicate bath composed of 2 g/L NaOH and 12 g/L Na_2_SiO_3_ was used, modified with Na_2_SiF_6_ or NaF; however, their amounts were not specified. The coating obtained in the electrolyte containing Na_2_SiF_6_ exhibited a smoother surface morphology than those processed in the presence of NaF. In the presence of Na_2_SiF_6_ in the electrolyte, the coating was slightly thicker than the one obtained with NaF (45 µm in comparison to 40 µm) and contained a lower number of internal pores. Both coatings were composed of Mg_2_SiO_4_ and MgO, and their molar ratio was approximately equal to 1.1. The layer produced in the electrolyte containing Na_2_SiF_6_ showed a higher hardness (~1150 HV) and a lower wear rate (~3 µg·N^−1^·m^−1^) compared to that modified with NaF (~950 HV and ~4 µg·N^−1^·m^−1^, respectively).

The investigation of the effect of Na_2_SiF_6_, in the range of 0.3–0.7 g/L, on the mechanical properties of the coatings produced on AZ31B alloy in an alkaline silicate bath (2 g/L NaOH and 12 g/L Na_2_SiO_3_) has been continued in [120]. The coating obtained with the addition of 0.5 g/L Na_2_SiF_6_ exhibited the highest thickness (~85 µm) and hardness (~12 GPa), as well as the lowest wear loss (~3 µg·N^−1^·m^−1^) and porosity (~3%). This behavior was attributed to an increase in the number of fine sparks. A further increase in Na_2_SiF_6_ concentration resulted in the formation of large destructive discharges. XRD analysis showed the presence of MgO, MgF_2_, and Mg_2_SiO_4_ phases within the coatings formed in the presence of Na_2_SiF_6_. The absence of silica peaks in the XRD pattern confirms its amorphous form. The amount of MgF_2_ initially increased to the concentration of Na_2_SiF_6_ equal to 0.5 g/L and then decreased. This drop was suggested to be caused by the conversion of an excess of Na_2_SiF_6_ to SiF_6(g)_ during the PEO conditions. It was also indicated that dissociation of Na_2_SiF_6_ at the beginning of the PEO process produces MgF_2_ and Mg_2_SiO_4_ (Equation (22)), which play a key role in determining the mechanical and tribological properties of the coatings.

SiF_6_^2−^ + 2Mg^2+^ + 2OH^−^ → MgF_2_ + Mg_2_SiO_4_ + H_2_O (22)

The following article in the series [121] has described the influence of the PEO process time (5–60 min) on the properties of the coatings formed on AZ91D alloy in an alkaline bath (3 g/L NaOH + 12 g/L Na_2_SiO_3_) containing 0.3 g/L Na_2_SiF_6_. The increase in treatment time from 5 to 60 min was found to improve the coating thickness (from ~20 to ~150 µm) and hardness (from ~700 to ~1300 HV). The coating anodized for 15 min exhibited the most compact and defect-free intermediate layer structure. Based on EDS analysis, the presence of O, Mg, Si, F, Al, and Na was detected; however, the fluorine content was relatively small, it changed from 0.23 to 0.92 at.% for 5–60 min. XRD studies indicated the presence of MgO, Mg_2_SiO_4_, and MgF_2_ phases. Corrosion tests (PDP in 3.5 wt.% NaCl) demonstrated the best resistance for the coating produced for 60 min, which was attributed to its highest thickness and the highest amount of MgF_2_ incorporated into the layer.

Optimization of the Na_2_SiF_6_ content (0.1–0.7 g/L) in an alkaline silicate bath (3.5 g/L NaOH + 12 g/L Na_2_SiO_3_) has also been performed [122] for the PEO of AZ61 alloy. The process was carried out for 30 min under the same electrical regime as in previous articles [114,115,116]. The best mechanical properties (hardness and wear rate) as well as corrosion resistance showed the coating prepared with the 0.3 g/L addition of Na_2_SiF_6_. The coatings obtained using larger amounts of the additive contained more pores and defects within the inner layer, which weakened their barrier properties.

The influence of the presence of Na_2_SiF_6_ (0.0077 mol/L = 1.45 g/L), NaF (0.046 mol/L = 1.93 g/L), or LiF (0.046 M = 1.19 g/L) in an alkaline silicate bath (0.15 mol/L KOH + 0.086 mol/L Na_2_SiO_3_) on the properties of PEO coatings formed on AZ31 alloy using bipolar current in galvanostatic mode (*j* = 17.5 A/dm^2^, *U*_+_ = 400 V, *U*_−_ = −30 V, *f* = 67 Hz, *D* = 66.7%, *t* = 4 or 30 min) has been described [78]. The baths exhibited a comparable strongly alkaline pH of approximately 13.8 and differed only slightly in conductivity, which was equal to 45.4, 43.78, 43.40, and 43.33 mS/cm for the addition of LiF, NaF, Na_2_SiF_6_, and the fluoride-free bath, respectively. In the case of the 4 min synthesis, the coating obtained in the electrolyte without fluorides was characterized by the most compact outer layer and was thicker (8.5 µm) than those produced with fluoride additives (7.1, 7.2, and 5.0 µm for LiF, NaF, and Na_2_SiF_6_, respectively). The properties of the coatings obtained after 30 min were different. The thinnest layer was formed in the base bath (11.8 µm), followed by slightly thicker layers with the addition of NaF (13.4 µm) and Na_2_SiF_6_ (15.9 µm). The layer obtained from the bath containing LiF was clearly the thickest (25.5 µm). All layers had a structure with closed internal pores. The coatings obtained in 30 min were characterized by the higher amount of F and Mg incorporated, as well as the lower content of Si and O. EDS analysis indicated that all coatings had a similar amount of F in the inner layer (approximately 5.5 at.%). Interestingly, no increase in the amount of Si incorporated was observed for the bath containing Na_2_SiF_6_. XRD analysis did not show any peaks for MgF_2_, suggesting that fluorine was present in an amorphous state or that its concentration was below the detection limit of the applied method. For coatings modified with NaF and Na_2_SiF_6_, an increased amount of MgO was recorded. It was demonstrated (EIS and immersion tests in a 0.1 M NaCl solution saturated with Mg(OH)_2_ and in PBS) that the coating formed in the presence of the NaF additive had the best protective properties, followed by the one that contained Na_2_SiF_6_ and LiF. This behavior was related to the total amount of fluorine incorporated into the coatings, which reached 2.8, 2.5, and 1.6 at.% (after 4 min of PEO) and 6.1, 5.6, and 5.0 at.% (after 30 min of PEO) for modified coatings with NaF, Na_2_SiF_6_, and LiF, respectively.

#### 4.2.4. AlF_6_^3−^

Another example of a complex fluorine salt that has been applied in the PEO synthesis of magnesium alloys is Na_3_AlF_6_. The properties of a conversion coating formed with the addition of Na_3_AlF_6_ (5–10 g/L) to an alkaline phosphate bath (5 g/L KOH + 5–15 g/L (NaPO_3_)_6_ + 4 mL/L TEA, pH = 11–12) have been described [123]. These results were compared with a coating synthesized in an alkaline silicate bath (3 g/L KOH + 6–15 g/L Na_2_SiO_3_·9H_2_O + 4 g/L Na_2_B_4_O_7_·10 H_2_O + 3 g/L KF·2H_2_O, pH = 13–14). The PEO syntheses were performed on AZ91D alloy using high frequency bipolar current mode (*f* = 700 Hz, *U*_−_ = 60 V, *D* = 30%) using a “step-down current method”, which made the PEO process more stable and more effective. The method used high current pulses 7–9 A/dm^2^ and 4–6 A/dm^2^ to reach anodic voltages *U*_+_ = 200 V, and then *U*_+_ = 360 V for the silicate bath and *U*_+_ = 420 V for the phosphate bath, and after that the current was kept at the level of 0.5–1 A/dm^2^. The coating obtained in the presence of Na_3_AlF_6_ was thinner (37 µm) and more homogeneous than the silicate-derived layer (62 µm). Within the inner barrier layer of both coatings, the presence of Mg, O, F, K, and minor amounts of other bath constituents was detected. The phosphate-derived coating showed a thicker fluorine-enriched zone (1–2 µm) compared to the silicate coating bath (0.7–1 µm). XRD analysis showed that the coatings were composed of MgO and MgAl_2_O_4_ or Mg_2_SiO_4_, depending on the bath used and the presence of an amorphous phase in both coatings. Corrosion tests (PDP, EIS, and immersion tests in a 5 wt.% NaCl) indicated good protective properties of both coatings, although no distinct advantage of one over the other was observed.

Subsequent research [124], using transmission electron microscopy with selected area electron diffraction (TEM-SAED), has demonstrated that fluorine was located near the substrate in both the inner and outer layers. The fluorine-rich region was primarily composed of amorphous material (no crystalline fluorine compounds such as MgF_2_ or NaMgF_3_ were detected), along with a small amount of nanocrystalline MgO (20–60 nm). Furthermore, enrichment in nanocrystalline MgO particles was observed within the inner layer.

#### 4.2.5. Other Complex Anions

In our research, we have used other complex fluoride salts such as NaSbF_6_ [125], NaBF_4_ [40], and KPF_6_ [126], in the PEO process on magnesium alloys. In the cases of the tetrafluoroborate and hexafluorophosphate salts, the properties of the resulting coatings were compared with those obtained in baths containing equimolar amounts of fluorine in the form of a simple salt (NaF). In both cases, the use of the complex salt enabled the incorporation of a higher amount of fluorine into the coating in comparison to the use of NaF under identical current conditions. Consequently, an improvement in the corrosion resistance of the coatings was recorded.

## 5. Discussion

Fluorine compounds, in the form of both simple and complex ions, are frequently used additives in electrolytic baths for the anodic formation of conversion coatings on magnesium alloys. Initially, aggressive baths based on hydrofluoric acid and its salts were used. With technological advancements, various types of electrolytes containing fluoride salt additives have been implemented. Subsequently, low-voltage processes were replaced by the PEO process, and the electrolytes were enriched with complex fluoride salts. In the latest patents, a trend of combining simple and complex fluorides with organic additives can be observed.

The introduction of simple fluoride salts (KF and NaF) into the electrolyte leads to an increase in solution conductivity, thereby altering the intensity of electrical discharges. When galvanostatic conditions are applied, a decrease in the breakdown voltage value occurs. When using direct current (DC) and pulsed (monopolar or bipolar) signals, the most commonly observed effect is an increase in conversion coating thickness, accompanied by a decrease in the number of surface pores and a simultaneous increase in their diameter. This behavior can be directly correlated with an increase in the intensity of discharges that occur on the surface during the PEO process. The different behavior reported in [69] can be explained by the applied alternating current (AC) signal, which introduces a large cathodic signal (*i*_a_/*i*_c_ = 1), which significantly affects the magnesium anodization process.

Increasing the fluoride concentration in the electrolyte results in greater fluorine incorporation into the newly formed coating. However, the efficiency of fluorine incorporation depends on both the applied electrical regime and the other components of the bath. For example, the introduction of 8 g/L KF into an alkaline silicate bath used in the oxidation process under DC current at a voltage of 300 V allowed the incorporation of only 3 at.% fluorine [70], while the introduction of 11.6 g/L KF in the synthesis performed under constant current density (5 A/dm^2^) allowed the incorporation of 13.5 at.% fluorine [72]. One of the highest reported fluorine contents was 25.74 wt.% (28.5 at.%) in a coating produced using a bipolar pulsed signal (with an unknown current density) from an alkaline electrolyte containing (NaPO_3_)_6_ and 15 g/L KF [70]. Interestingly, despite such a large amount of fluorine incorporated, the coating obtained demonstrated biocompatibility and the absence of cytotoxicity for model cells [71,72].

Most XRD analyses confirm the presence of a crystalline MgF_2_ phase in the synthesized conversion coatings, which can directly explain the increase in the mechanical properties of the coatings [69,73]. However, it has been reported [74] that despite a high fluorine content (20 at.%), the presence of a crystalline MgF_2_ phase was not found, suggesting that fluorine can also occur in an amorphous form. Interestingly, in this study, a disproportionate distribution of fluorine was also observed throughout the coating cross-section, with its increased amount in the inner layer (close to the magnesium substrate), which is frequently emphasized in recent research, especially in the context of corrosion resistance [40,75,78].

Although an increase in the protective properties of conversion coatings is generally observed with increasing fluoride concentration in the electrolyte, it was also shown [74] that even a small amount of fluoride salt (0.02–0.05 mol/L KF; ~1–3 g/L) in the electrolyte causes a significant increase in coating thickness and improves the anti-corrosion properties. This was confirmed in [75], where the formation of a thin fluoride-containing nanolayer was found using HAADF-STEM measurements (on the nanoscale) at amounts greater than 0.8 g/L NaF, which directly improves corrosion resistance.

KF and NaF are the most commonly used simple fluoride salts in PEO of magnesium alloys. However, the influence of NH_4_F, LiF, and CaF_2_ was also investigated. A direct comparison of the effects of KF and NaF indicated better properties of the coating produced in the presence of KF, which was explained by the higher mobility of K^+^ ions compared to that of Na^+^ [76]. In turn, investigations of the influence of NH_4_F demonstrated a more beneficial effect of this salt on anti-corrosion properties compared to NaF [77]. The presence of NH_4_F caused a decrease in both the conductivity and pH of the solution. For the coatings obtained in the presence of NH_4_F, a higher amount of MgF_2_ incorporated into the coating was observed, which was explained by a change in the precipitation equilibrium of this product compared to MgO due to a change in the pH of the electrolyte (a lower amount of OH^−^). However, the application of LiF did not produce results as good as those of NaF, which was related to the poor solubility of the LiF salt [78]. In contrast to this argument, the addition of CaF_2_ showed that despite its highly limited solubility, fluorine was still integrated into the conversion layer, improving corrosion resistance compared to the coating without fluorine [79]. These observations suggest that CaF_2_ could be incorporated into the coating via a mechanism similar to the addition of other nanoparticles in the form of a colloidal solution through their deposition on the coating surface and melting at high temperatures during the PEO process. In this case, adequate stirring of the system is necessary to prevent sedimentation of undissolved particles. Investigations on the role of KF in an acidic electrolyte explicitly demonstrated that the presence of dihydrogen phosphate substantially improved fluorine incorporation [80]. Therefore, it can be concluded that the cation of the applied salt plays a significant role in the PEO process of magnesium alloys conducted in the presence of simple fluoride ions, directly affecting the conductivity and pH of the electrolytic bath, which consequently can alter both the intensity of the electrical discharges and the tendency to form specific products.

Among the complex fluoride salts, K_2_ZrF_6_ was the additive investigated the most. The presence of this compound allowed the formation of coatings containing various forms of ZrO_2_ (tetragonal, cubic, monoclinic), Mg_2_Zr_5_O_12_, and MgF_2_, which increased their hardness and corrosion resistance. Initially, PEO syntheses were carried out in an acidic environment most often containing a mixture of NaH_2_PO_4_ and H_3_PO_4_ [81,82]. The low pH of the solution significantly reduced the amount of MgO incorporated, promoting the formation of other phases instead, which improved the barrier performance of the layers. However, as a result of the aggressiveness of the acidic medium toward the magnesium substrate, it was necessary to introduce electrolyte additives to decrease this effect [83,84,85,86] or implement a two-step process that involved initial passivation in an alkaline bath [87,88,92]. The presence of phosphates and organic compounds (citrate, triethanolamine) limited substrate corrosion, while the addition of a simple fluoride salt caused rapid surface passivation with an MgF_2_ layer, which restricted magnesium dissolution during the first stage of the synthesis (without discharges) and led to pore sealing (also through MgF_2_ formation) in the plasma stage (with discharges). A direct comparison of the coatings produced in the acidic bath containing K_2_ZrF_6_ with the coatings synthesized in a conventional alkaline bath (without the addition of a fluorine source) showed better anti-corrosion properties for the K_2_ZrF_6_-derived coatings [90]. The morphology of these coatings was different; the coatings produced in the acidic bath with K_2_ZrF_6_ had three layers [82,83,88,89,92]. This is a clear change compared to the two-layer coatings produced in alkaline baths with simple fluorides. In the case of three-layer coatings, very large amounts of fluorine were recorded in all layers (even up to approximately 50 at.% [82]) together with an exceptional outer layer composed of hard ZrO_2_ with pores sealed by MgF_2_, which provided an improvement in mechanical and anti-corrosion properties. However, it should be emphasized that the presence of the complex salt K_2_ZrF_6_ was not always sufficient to provide an adequate amount of fluoride to effectively seal the outer pores, which is why the baths were enriched with an additional source of fluorine in the form of KF or NaF [83,84,85,86]. Regarding K_2_ZrF_6_ as a zirconium source, it was confirmed that this salt provides better coating properties compared to alternative zirconium-containing salts (Zr(NO_3_)_4_ and ZrOCl_2_) [91]. However, the introduction of zirconium in the form of ZrO_2_ nanoparticles caused a completely different effect than the addition of K_2_ZrF_6_, which is soluble in an acidic environment [98,99]. Alkaline solutions (phosphate and silicate) represented an alternative type of bath containing K_2_ZrF_6_ [92,93,94]. The coatings obtained from strongly alkaline baths with K_2_ZrF_6_ exhibited a two-layer structure, analogous to that obtained in alkaline baths with simple fluorides. It should be stressed that the addition of K_2_ZrF_6_ drastically reduced the pH of the electrolyte, which, exceeding a critical value, modified the behavior of plasma discharge or completely suppressed it [93,94]. In reports describing the addition of K_2_ZrF_6_ in an alkaline environment, the formation of a precipitate in the bath due to the hydrolysis of ZrF_6_^2−^ ions was also indicated [95,96].

Analysis of the composition and characteristics of K_2_ZrF_6_ electrolytes, including pH values, requires careful consideration. Depending on the pH range in aqueous media, K_2_ZrF_6_ undergoes varying degrees of hydrolysis and exhibits different speciation [127], thus affecting its mechanism of incorporation into the conversion layer during PEO. In an acidic environment (pH < 3), the dominant form is the ZrF_3_^+^ cation. Increasing the pH to 4–5 promotes the presence of ZrF_6_^−^ anions. Meanwhile, within the pH range of 6–10, solid ZrO_2_ becomes the dominant phase, which can hydrate to (Zr(OH)_4_/ZrO_2_·nH_2_O). In highly alkaline media (pH > 11), anionic species such as Zr(OH)_5_^−^ can be generated.

Various additives have been used to stabilize the alkaline K_2_ZrF_6_ electrolytes: NaH_2_PO_4_ and trisodium citrate (typically used in acidic baths), (NaPO_3_)_6_ with TEA, as well as simple fluoride salts. The presence of polyphosphates alone can lead to the complexation of metal ions [128]. The combination of (NaPO_3_)_6_ and TEA was found to effectively suppress precipitation in the alkaline K_2_ZrF_6_ electrolyte during PEO of aluminum alloys [129]. It was suggested that these compounds could adsorb on the surface of Zr(OH)_4_ particles, acting as surfactants. Another crucial aspect dealt with the application of simple fluoride additives. First, they could restrict the hydrolysis of ZrF_6_^2−^ ions, shifting the equilibrium in favor of the complex salt form (Le Chatelier’s principle). Second, they provided an additional source of fluorine, sufficient to form a dense layer of MgF_2_ before the beginning of plasma discharges during PEO.

Therefore, not only the amount of K_2_ZrF_6_ in the bath is a critical parameter, but also the composition of the electrolyte. The presence of other components affects the pH, conductivity, complex formation ability, and stabilization of the ions. Consequently, this influences the mechanism of salt incorporation into the coating, which can lead to a different distribution of individual elements. It is worth noting that higher amounts of incorporated fluorine and zirconium are observed in the acidic environment.

K_2_TiF_6_ represents another complex salt used for the PEO of magnesium alloys. In the presence of this additive, the process has been carried out exclusively in conventional alkaline baths. It was demonstrated that the addition of 10 g/L K_2_TiF_6_ to an alkaline phosphate bath changes the character of the coating from a two-layer to a three-layer structure (as in the case of acidic baths with K_2_ZrF_6_) and allows the introduction of titanium (in the outer layer) and fluorine (mainly in the inner layer and within the pore-filling regions) [106]. This leads to an improvement in anti-corrosion properties [106,107,108] and increased biocompatibility [109]. However, it was observed that the introduction of K_2_TiF_6_ into the solution caused (in the first few hours) a substantial decrease in pH (from 11–12 to 6–7 after the introduction of 8–12 g/L of K_2_TiF_6_) with the formation of a white precipitate [110,111,112]. Therefore, it is worth noting that PEO synthesis is often ultimately performed under slightly acidic conditions. On the other hand, maintaining a high pH can be forced by introducing a large amount of a base [113,114]. This is significant because, for pH ≥ 6, hydrolysis of TiF_6_^2−^ ions occurs, leading to the formation of colloidal particles of TiO_2_ or Ti(OH)_4_ [130,131]. Therefore, in many cases, baths containing K_2_TiF_6_ are enriched with stabilizing additives (polyphosphates, TEA, simple fluorides), whose action is not always clearly explained [110,111,112,113]. It has been found [132] that during aluminum oxidation in the presence of K_2_TiF_6_ (4 g/L), the addition of (NaPO_3_)_6_ and TEA could limit particle agglomeration and precipitate formation due to adsorption on the surface of TiO_2_. However, it was indicated [114] that precipitate formation could also be restricted by applying an appropriate mixing sequence of bath components. The addition of K_2_TiF_6_ is advantageous because it leads to the formation of titanium phase-rich coatings (Mg_2_TiO_4_, TiO_2_, and Ti_2_O_3_) that allow the use of their potential biomedical and photocatalytic applications.

Another example of a complex fluoride salt used in the PEO of magnesium alloys is Na_2_SiF_6_. This compound is unstable in solutions with pH > 5, undergoing complete hydrolysis to Si(OH)_4_ and F^−^ (Equation (23)), which, due to the use of Na_2_SiF_6_ in drinking water treatment systems, has been thoroughly confirmed (among others, by ^19^F-NMR spectroscopy) [133,134].

SiF_6_^2−^ + 4H_2_O → 4H^+^ + 6F^−^ + Si(OH)_4_ (23)

The addition of 0.3 g/L Na_2_SiF_6_ to the electrolyte during the PEO of magnesium alloys has been used only in alkaline baths [119,120,121,122], which means that it is completely hydrolyzed under experimental conditions. The coatings produced contained Mg_2_SiO_4_ and MgF_2_ phases, and the improvement in mechanical and anti-corrosion properties was proportional to the amount of fluorine incorporated. However, the fluorine content in the coating was relatively low, up to 0.92 at.% [121]. The amount of fluorine ions released during complete hydrolysis of 0.3 g/L of Na_2_SiF_6_ corresponds to that present in 3.06 g of KF. When comparable amounts of KF were present in the alkaline electrolyte, the fluorine content in the coatings formed was also relatively low; for example, for 2–8 g/L KF it was in the range of 1–3 at.% [70] and for 2.9 g/L KF it was equal to 1.85 at.% [72]. The influence of compounds that stabilize the SiF_6_^2−^ ions in the electrolyte has not been investigated for the PEO of magnesium. However, such data were presented for aluminum PEO in an alkaline aluminate electrolyte (1 g/L KOH +3 g/L NaAlO_2_) containing 3 g/L K_2_SiF_6_ [135]. The presence of 10 g/L sodium citrate, which prevented the hydrolysis process of SiF_6_^2−^ in the bath, was shown to cause a substantial increase in the fluorine content of the coating, from 6.9 to 19.3 at.%, (recalculated from the values reported in wt.%) compared to the bath without this additive. It is interesting that the amount of silicone in the coating also increased from 0.8 to 5.8 at.%. It is suggested that the presence of citrate prevented the dissociation of SiF_6_^2−^ ions and that they can enter the electrochemical double layer on the metal surface. Only then could they decompose to silicon dioxide and fluorine ions at the high temperature of the PEO process (Equation (24)), increasing the contents of fluorine and silicon in the coating.

SiF_6_^2−^ + 2H_2_O → SiO_2_ + 4H^+^ + 6F^−^ (24)

It should be noted that 0.3 g/L Na_2_SiF_6_ was always used as the base electrolyte component when the effects of K_2_ZrF_6_ addition in alkaline media [95,96,97,98] were investigated, which could suggest that, being less stable (and thus acting as a source of fluorine), it served as a stabilizer for the ZrF_6_^2−^ ion.

Only a few studies have shown a positive effect of Na_3_AlF_6_ on the corrosion resistance of coatings formed on magnesium alloys [123,124]. Importantly, the TEA additive was used in both cases, and TEM-SAED analyses confirmed the incorporation of fluorine in both the inner and outer layers. When considering Na_3_AlF_6_ as an electrolyte component in a PEO process, the possibility of its complete hydrolysis at pH > 8 with the release of simple F^−^ ions (which was confirmed by the ^19^F NMR spectra) should be taken into account [136]. The composition of the coating produced with the addition of Na_3_AlF_6_ showed the presence of crystalline MgO and MgAl_2_O_4_, as well as a small amount of amorphous fluoride compounds. However, the presence of stabilizers, such as potassium tartrate [137] or TEA [138], in the electrolyte inhibited the hydrolysis of AlF_6_^3−^, as in the case of SiF_6_^2−^, thereby enabling the intact AlF_6_^3−^ ion to react under the high-temperature conditions of the plasma discharge to form Al_2_O_3_ (Equations (25)–(27)) and uniformly incorporate fluorine-containing phases into the coating [138].

AlF_6_^3−^ + 3H_2_O → Al(OH)_3_ + 3H^+^ + 6F^−^ (25)

Al(OH)_3_ → *γ*-Al_2_O_3 _ (T~723K) (26)

*γ*-Al_2_O_3_ → *α*-Al_2_O_3 _ (T~1373K) (27)

Taking into account the stability of complex fluoride salts in an alkaline environment (most commonly used in the PEO of magnesium), hexafluorophosphate appears to be an interesting additive [139]. Early studies on this additive have demonstrated that the use of 2.5 g/L of KPF_6_ in an alkaline silicate bath allowed the incorporation of more fluorine and consequently improved the anti-corrosion properties, compared to the equimolar amount of NaF. Taking into account the improvement of mechanical properties, the use of a hexafluorohafnate salt (HfF_6_^2−^), also appears to be potentially interesting, which can potentially form hard HfO_2_ (analogously to ZrO_2_ from K_2_ZrF_6_).

**Figure 2:**
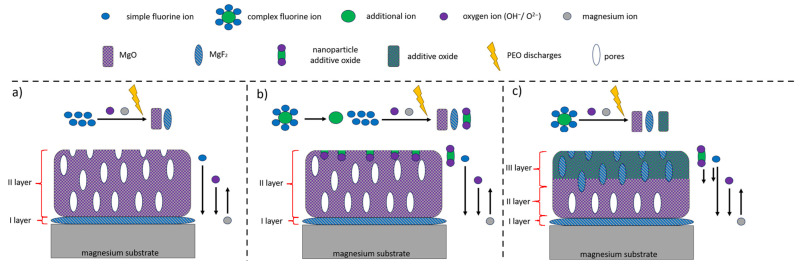
Mechanism of ions incorporation into PEO coating: (**a**) simple fluoride ion, (**b**) hydrolyzed complex fluoride ion, and (**c**) non-hydrolyzed complex fluoride ion.

Complex fluoride salts are generally unstable under alkaline conditions, undergoing hydrolysis to release simple fluoride ions and form colloidal oxides, such as ZrO_2_ and TiO_2_. Inhibition of this process (by lowering the pH value or introducing stabilizers) changes the character of the coating from a two-layer structure to a three-layer structure (sandwich-like system) and increases the amount of fluorine incorporated, also in the new outer layer, which was indicated in the case of the K_2_ZrF_6_ additive. Therefore, the use of a lower pH for synthesis with K_2_TiF_6_, K_2_SiF_6_, and Na_3_AlF_6_, together with strategies to prevent the rapid decomposition of these complex salts (e.g., by introducing stabilizers) and to ensure that the intact ions react only during the plasma stage, represents a promising direction for future research. The scheme of incorporation of simple and complex fluoride ions, taking into account the form (hydrolyzed or non-hydrolyzed) of complex ions, is shown in Figure 2.

In PEO synthesis on magnesium alloys in an electrolyte containing simple fluorides, MgF_2_ is formed mainly in the barrier layer of the coating (the inner layer). When a complex fluoride salt is present in the electrolyte, the incorporation mechanism differs depending on whether the entire ion or its hydrolysis products react in the PEO process. If the complex ion undergoes hydrolysis, the incorporation mechanism is similar to that of the reaction with simple fluorides. Fluorine is incorporated mainly into the inner layer; however, colloidal particles, usually oxides, formed from additional complex salt elements (e.g., Zr and Ti), are deposited on the surface of the outer layer and can partially melt and combine with the forming MgO layer. However, if the complex ion does not undergo hydrolysis in the electrolyte before PEO (due to a pH-induced shift in equilibrium and/or the use of stabilizers), it decomposes only during the high-temperature plasma stage. As a result, fluorine is present both in the barrier layer and in the pores of the outer layer. In addition, the other elements of the complex salt melt and mix to a greater extent with the elements that form the outer layer. A sandwich-type (three-layer) coating is formed.

## 6. Conclusions

Fluorine-containing compounds represent one of the most common classes of electrolyte additives used in PEO of magnesium alloys. The presence of simple fluoride ions enhances the electrolyte conductivity, directly leading to a lower breakdown voltage and an increase in the layer thickness. Furthermore, the early formed MgF_2_ suppresses the dissolution of the magnesium substrate during the non-discharge stage and is subsequently integrated into the conversion layer during the plasma process, improving both mechanical and corrosion performance of the coating formed.

Among the complex fluoride salts, K_2_ZrF_6_ is the additive applied most frequently, both in acidic and alkaline baths. Its presence leads to the incorporation of MgF_2_, as well as ZrO_2_ and Mg_2_Zr_5_F_12_, which improves both the hardness and anti-corrosion properties of the coating formed. In many cases, instead of a two-layer structure, a three-layer coating is obtained with an outer layer sealed with ZrO_2_.

The use of the K_2_TiF_6_ additive leads to the formation of MgF_2_, as well as TiO_2_ and Mg_2_TiO_4_. The presence of TiO_2_ improves the biocompatibility of coatings and offers possible photocatalytic applications. However, this additive is less stable than K_2_ZrF_6_, leading to precipitate formation and a distinct change in pH of the original alkaline electrolyte solution.

Na_2_SiF_6_ and Na_3_AlF_6_ are quite unstable in alkaline solutions and undergo hydrolysis almost immediately, releasing simple F^−^ ions.

Keeping the complex fluoride ions intact during the PEO process can lead to a higher fluorine content in the layers formed and the improvement in the properties of the resulting layers. Therefore, the degradation of the complex fluoride salt should be prevented by the presence of chelating agents and/or simple fluoride salts in the baths.

Considering the potential of complex fluoride salts in PEO electrolytes, it would be worthwhile investigating previously unexplored or little-studied complex fluoride ions, such as PF_6_^−^ and HfF_6_^2−^.

## Figures and Tables

**Table 1:** Summary of major patents concerning the electrolytic formation of conversion coatings on magnesium substrates utilizing fluoride-containing electrolytes.

Inventor (Application Filed by/Technology Name)	Patent Number	Date of Patent	Composition of the Solution	Electrical Conditions	Ref.
Keeler L.J. (American Magnesium Corp., Niagara Falls, NY, USA)	US 1,574,289	1926	-HF	DC *U* = 110 V	[46]
Budiloff N., Schnabel W.	US 2,305,669	1942	-NaAlO_2_ -NaF -Na_3_PO_4_ -Na_3_AsO_4_	DC/AC *j* = 1–3 A/dm^2^	[47]
Loose W.S. (Dow Chemical Company, Midland, MI, USA)	US 2,313,753	1943	-HF/NaF/KF/ H_2_F_2_/NaHF_2_/KHF_2_/NH_4_HF_2_/ /H_2_SiF_6_/HBF_4_	DC *U* = 100 V	[48]
Buzzard R.W.	US 2,314,341	1943	Alkaline solution and one or more from the group: -chromates -dichromates -permanganates -borates -silicates -sulphates -fluorides (NaF/KF/NH_4_F/Na_2_SiF_6_) -phosphates -molybdates	DC/AC *j* = 0.5–100 A/ft^2^ *U*_max_ = 150 V	[49]
Evangelides H.A. (HAE)	US 2,723,952	1955	-KOH -Al(OH)_3_ -KF -Na_3_PO_4_ -K_2_MnO_4_	DC/AC *j* = 10–20 A/ft^2^ *U*_max_ = 85 V	[50]
Delong H.K. (Dow Chemical Company)	US 2,901,409	1959	-NH_4_HF_2_/NaHF_2_ -Na_2_C_2_O_7_·2H_2_O -H_3_PO_4_	DC/AC *U* = 120 V	[51]
Kozak O.	US 4,620,904	1986	Alkaline solution with addition of a source: -silicate (K_2_SiO_3_/Na_2_SiO_3_/Li_2_SiO_3_/ K_2_SiF_6_), -fluoride (HF/NaF/KF/H_2_SiF_6_)	DC *U* = 150–400 V	[52]
Kobayashi W. et al. (Ube Industries Ltd., Ube, Japan)	US 4,744,872	1988	Alkaline solution with: -silicate, -carboxylate -phosphate -fluoride (LiF/NaF/KF)	AC *j* = 0.2–5 A/dm^2^	[53]
Schmeling E.L. et al. (Electro Chemical Engineering GmbH, Zug, Switzerland)	US 4,976,830	1990	Alkaline solution with: (a) borate/sulfonate anions and (b) phosphate and fluoride (HF/H_2_F_2_) or chloride ions	DC + AC *f* = 10–100 Hz *D* = 15–30% *j* = 1–6 A/dm^2^ *U*_max_= 100 V	[54]
Bartak D.E. et al. (Technology Applications Group, Grand Forks, ND, USA/Tagnite)	US 5,240,589	1993	1st step (chemical): NH_4_F 2nd step (electrochemical): alkaline silicate solution with: -fluorides (NaF/KF/NH_4_F/LiF/RbF/CsF/HF) or fluorosilicate (Na_2_SiF_6_/K_2_SiF_6_/Li_2_SiF_6_)	pulse DC *j* = 0.2–9 A/dm^2^ *U*_max_ = 100–400 V	[55]
Kurze P. et al. (Electro Chemical Engineering GmbH/Magoxid-Coat)	US 5,385,662	1995	-phosphate ions, -borate ions -fluoride ions with addition of: urea/hexamethylenediamine/hexamethylenetetramine/ethylene glycol/glycerin	DC/AC/3-phase current/pulse current *j* > 1 A/dm^2^ *U*_max_ = 400 V	[56]
Barton T.F. (Magnesium Technology Limited, Auckland, New Zealand/Anomag)	US 5,792,335	1998	-NH_4_OH -phosphate (Na_2_HPO_4_/NH_4_NaHPO_4_/NH_4_H_2_PO_4_/(NH_4_)_2_HPO_4_) alternative additive: NaF and NaAlO_2_	DC/AC *U* = 350 V	[57]
Dolan S.E. (Henkel Corporation, Düsseldorf, Germany)	US 2003/0075453	2003	-phosphate -permanganate -silicate -zirconate -titanate -complex fluoride (Ti/Zr/Hf/Si/Sn/Al/Ge/B (acids and salts)	pulse DC *U* = 150 V	[58]
Yun G.Y et al. (Wiscohitec Co., Ltd., Gimcheon, Republic of Korea)	US 8,337,689	2012	-NaOH -NaF -Na_3_PO_4_ -Na_4_P_2_O_7_ -Al(OH)_3_ -Na_2_SiF_6_ -potassium acetate -rare earh metal powder	not specified	[59]
Liu F. et al. (Institute of Metal Research of CAS, Shenyang, China)	CN 101,994,145	2012	-fluorozirconate ((NH_4_)_2_ZrF_6_/K_2_ZrF_6_/Na_2_ZrF_6_) -dihydric phosphate (NH_4_H_2_PO_4_/KH_2_PO_4_/NaH_2_PO_4_) -fluoride (KF/NaF/NH_4_F/NH_4_HF_2_ -citrate (K/Na/NH_4_)	*U* = 350–420 V *f* = 100–1000 Hz *D* = 20–60%	[60]
Wang F. et al. (Institute of Metal Research of CAS)	CN 102,560,600	2014	-NaOH/KOH -Na_2_CO_3_ -urea -Na_2_SiF_6_/Na_3_AlF_6_/NaBF_4_/ Na_2_ZrF_6_ -NaF	DC/AC *U* = 20–650 V *f* = 20–3000 Hz	[61]
Bonheun G. et al. (Changwon National University, Industry-Academic Cooperation Foundation, Changwon, Republic of Korea)	KR 102,056,412	2019	-NaOH -CeO_2_ -Na_2_SiO_3_ -Na_2_SiF_6_	AC U = 150–250 V DC U = 200–400 V	[62]
Raga K. et al. (Pratt & Whitney, Rzeszów, Poland)	US 11,001,927	2021	Alkaline silicate solution with tetrafluoroborate (NaBF_4_/KBF_4_/LiBF_4_/NH_4_BF_4_)	pulse DC (mono-/bipolar) j = 0.1–20 A/dm^2^ U_max_ = 600 V	[63]
Zhang Y. et al. (Taiyuan University of Science and Technology, Taiyuan, China)	CN 112,899,753	2022	-silicates -cerium salts -fluoride (KF/NaF) -zirconium salts -sodium hydroxide -citric acid -acetates	pulse DC (bipolar) *U* = 300/−50 V *f* = 300–600 Hz *D* = 38–51%	[64]
Gao Z. et al. (12th Research Institute of China State Shipbuilding Corp. Co., Ltd., Xingping, China)	CN 120,818,872	Pending (Published in 2025)	-NaOH/KOH -NaAlO_2_/KAlO_2_ -fluoroaluminate (Na_3_AlF_6_/K_3_AlF_6_) -AlF_3_ -glycerol	pulse DC *j* = 1–5 A/dm^2^ *U*_max_ = 450–550 V	[65]
Guo Q. et al. (Institute of Metal Research of CAS)	CN 121,496,531	Pending (Published in 2026)	-NaOH -Na_2_SiO_3_ -Na_3_PO_4_ -NaF/KF -glycerol -acrylic emulsion Na_2_ZrF_6_	pulse DC *j* = 0.3–0.7 A/dm^2^ *f* = 5000–6000 Hz *D* = 45–50%	[66]

## Data Availability

The original contributions presented in this study are included in the article. Further inquiries can be directed to the corresponding authors.

## Abbreviations

The following abbreviations are used in this manuscript:

AC	Alternating Current
*D*	Duty Cycle
DC	Direct Current
EDS	Energy Dispersive X-ray Spectroscopy
EIS	Electrochemical Impedance Spectroscopy
*f*	Frequency
FNL	Fluoride-Containing Nanolayer
HA	Hydroxyapatite
HAADF-STEM	High-Angle Annular Dark-Field Scanning Transmission Electron Microscopy
HBSS	Hank’s Balanced Salt Solution
*j*	Current Density
*j*_corr_	Corrosion Current Density
Na_12_Phy	Sodium Phytate
PBS	Phosphate-Buffered Saline
PDP	Potentiodynamic Polarization
PEO	Plasma Electrolytic Oxidation
SAED	Selected-Area Electron Diffraction
SBF	Simulated Body Fluid
SEM	Scanning Electron Microscopy
*t*	Time
TEA	Triethanolamine
TEM	Transmission Electron Microscopy
TEM-SAED	Transmission Electron Microscopy with Selected Area Electron Diffraction
*U*	Voltage
*U*_max_	Maximum Voltage
XPS	X-ray Photoelectron Spectroscopy
XRD	X-ray Diffraction
